# Magnetoelectric Multiferroics for the Next‐Generation Ultralow‐Power Electronics

**DOI:** 10.1002/advs.78344

**Published:** 2026-10-18

**Authors:** Yang Liu, Yunlong Sun, Hao Luo, Duoduo Zhao, Yuhan Zeng, Xuzihan Zhang, Zhewei Liu, Hao Peng, Zhijing Zhang, Jack Yang, Ji Zhang, Danyang Wang, Junjie Shi, Sean Li

**Affiliations:** ^1^ Materials and Manufacturing Futures Institute School of Materials Science and Engineering The University of New South Wales Sydney NSW Australia

**Keywords:** efficient energy use, electronic engineering, electronics, magnetoelectric effect, materials science, multiferroics

## Abstract

Magnetoelectric multiferroics enable voltage control of magnetic states for low‐energy, non‐volatile memory and logic. Their practical value, however, depends on the complete device pathway, not coupling strength alone. This Review evaluates materials, switching mechanisms, and devices against these requirements, distinguishing measured performance from projected targets. We compare three magnetoelectric random‐access memory (MeRAM) routes with ferroelectric RAM and spin‐transfer torque magnetic RAM, and assess magnetoelectric spin–orbit (MESO) logic alongside complementary metal‐oxide‐semiconductor (CMOS) technology and current‐driven spintronics. MeRAM combines electric‐field writing, magnetic storage, and nondestructive readout, with prospects for low write energy and closer memory–logic integration. However, no integrated device has yet combined these benefits with competitive speed, retention, endurance, read margin, and density. For magnetoelectric logic, projected low‐voltage, non‐volatile operation has yet to be matched by experimental demonstrations of the full conversion path, gain and fan‐out. Results from BiFeO_3_, room‐temperature hexaferrites and other platforms identify deterministic switching, interface quality and thin‐film integration as central to closing the gap between projections and experiments. Magnetoelectric devices are therefore more likely to complement CMOS than replace it, particularly in logic‐in‐memory, compute‐near‐memory and normally‐off systems, where non‐volatility and reduced data movement could support next‐generation ultralow‐power electronics.

## Graphical abstract

Magnetoelectric coupling provides a unifying route from electric‐field control of magnetic order to non‐volatile MeRAM, MESO logic, and emerging antiferromagnetic and magnonic devices. By connecting switching physics with device benchmarks, material platforms, and integration strategies, distinctive technological opportunities are identified alongside the challenges that must be overcome to enable scalable, energy‐efficient computing technologies.

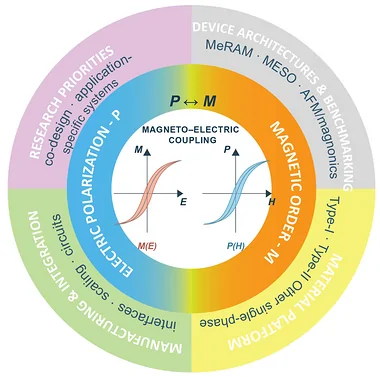

## 1 Introduction

Magnetoelectric multiferroics offer a promising route toward beyond‐CMOS electronics by enabling electric‐field control of magnetic order [1]. In these systems, coupling between ferroelectric and magnetic degrees of freedom allows magnetization to be manipulated by voltage rather than by current, providing a fundamentally different switching mechanism from charge‐based CMOS or current‐driven spintronics [2]. This concept is especially attractive for future logic and memory because it combines two desirable features that are difficult to achieve simultaneously in conventional platforms: non‐volatility and low switching energy.

The appeal of magnetoelectric switching arises from the growing limitations of CMOS scaling. As transistor dimensions approach the atomic scale, thermionic field‐effect transistors remain constrained by the Boltzmann limit of 60 mV dec^−1^ at room temperature, preventing indefinite reduction of the supply voltage (usually ∼700 mV) and contributing to the well‐known power wall. At the system level, this challenge is compounded by the memory wall, in which the energy and latency required to move data between separated logic and memory increasingly dominate the overall computing cost. Together, these bottlenecks have motivated the intense interest in formulating new electronic device concepts that go beyond charge‐based switching and can support tighter integration of logic and memory.

In this context, magnetoelectric multiferroics are particularly compelling because they offer voltage‐controlled magnetism without the Joule‐heating penalty associated with spin‐transfer torque or spin‐orbit torque. In principle, switching is governed primarily by capacitive charging and interfacial coupling, raising the prospect of extremely low write energy together with non‐volatile magnetic state retention. These attributes have made magnetoelectric phenomena central to the proposals for energy‐efficient spintronic architectures such as magnetoelectric spin‐orbit (MESO) logic, Magnetoelectric random‐access memory (MeRAM), and related logic‐in‐memory concepts.

As highlighted in the timeline in Figure 1, the field has evolved from fundamental demonstrations of electric‐field control of magnetism to the development of increasingly device‐relevant and circuit‐aware platforms. Early milestones established the fundamental feasibility that multiferroic coupling could be used to influence ferromagnetism and exchange bias, while later studies demonstrated manipulable room‐temperature switching in engineered heterostructures. More recent work has expanded toward benchmarking, readout schemes, nanoscale integration, and system‐level proposals, reflecting a broader transition from proof‐of‐concept materials physics to candidate computing technologies.

**FIGURE 1:**
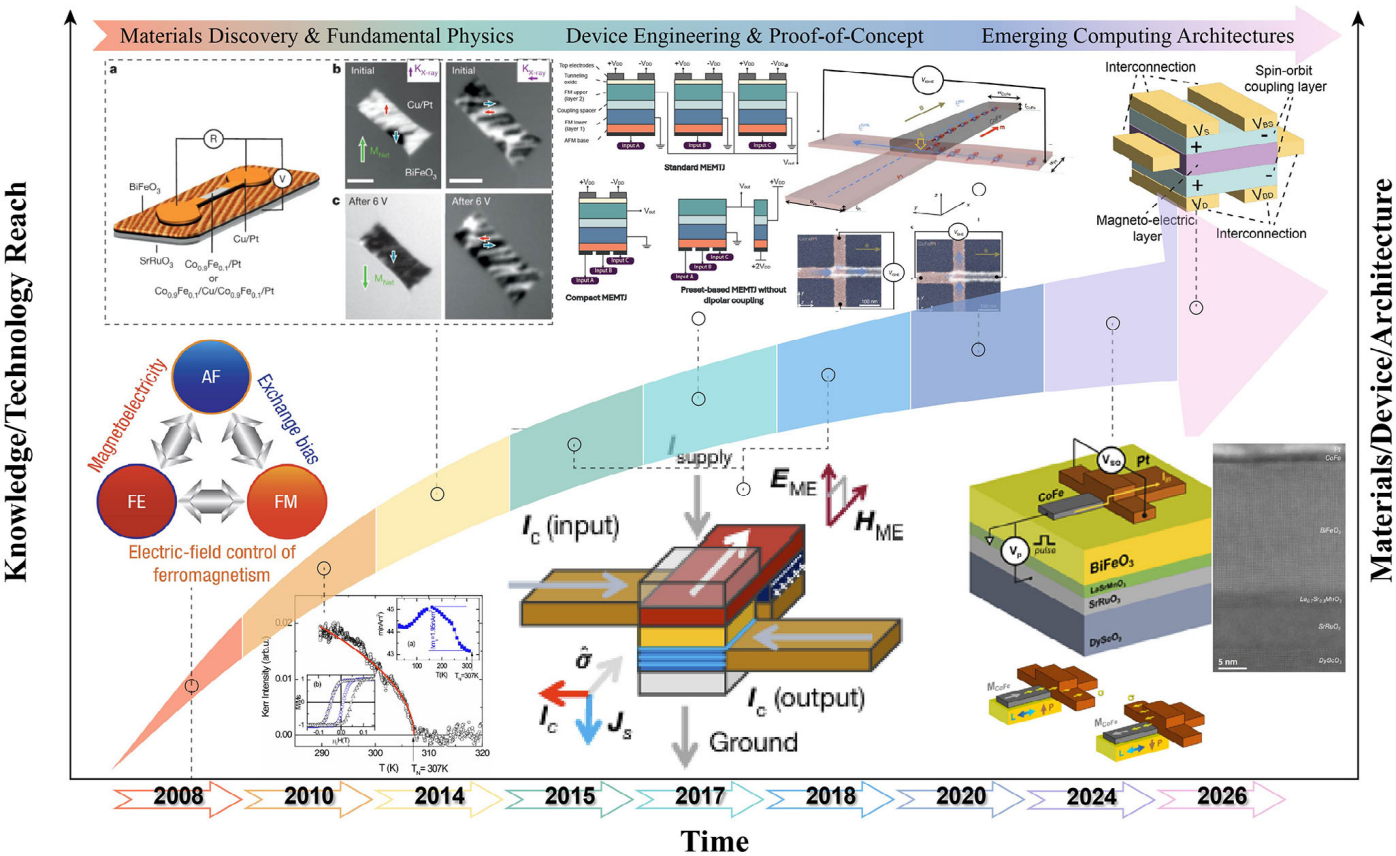
Evolution of magnetoelectric multiferroics for beyond‐CMOS logic (2008–2026). Key milestones in the development of magnetoelectric (ME) devices from fundamental materials discovery to circuit‐level concepts. Early studies demonstrated electric‐field control of magnetism [3] and room‐temperature switching [4], along with control of exchange bias [5]. The focus then shifted to device benchmarking and logic applications, with voltage‐driven switching identified as a low‐energy pathway [6] and later extended to ME‐based logic and neuromorphic systems [7]. System‐level proposals such as MESO [8] introduced integrated spin–charge architectures, followed by experimental progress in readout [9] and device integration [10]. Recent work explores magnon‐based switching for further energy reduction [11]. The upward trend reflects the increasing knowledge/technology reach and the transition from materials‐level phenomena to system‐level device architectures.

The central challenge for magnetoelectric electronics is no longer whether electric fields can influence magnetic order, but whether this coupling can be made strong, reliable, and scalable in device‐relevant platforms. Practical magnetoelectric systems must operate at room temperature, exhibit reproducible coupling between polarization and magnetism, and remain compatible with thin‐film growth, nanoscale fabrication, and semiconductor processing. Single‐phase multiferroics, especially BiFeO_3_, have played a foundational role because they combine robust ferroelectricity with room‐temperature antiferromagnetism and have enabled landmark demonstrations of electrically controlled magnetization in exchange‐coupled systems. At the same time, the limitations of single‐phase compounds, including weak net magnetization and restricted coupling geometries, have motivated growing interest in composite and interfacial heterostructures, where exchange, strain, and elastic coupling can be cooperatively engineered to deliver multifunctionalities in a single device system.

Equally important, the promise of magnetoelectric electronics should not be assessed on materials novelty alone. Practical relevance depends on whether magnetoelectric devices can deliver competitive switching energy, delay, signal gain, scalability, endurance, and integration compatibility when benchmarked against advanced CMOS and other emerging technologies. Moreover, the path from fundamental magnetoelectric coupling to a manufacturable computing platform remains highly non‐trivial. It requires room‐temperature operation, strong and reproducible coupling in nanoscale heterostructures, low‐loss interfaces for signal transduction, reliable device‐to‐device cascading, and process compatibility with semiconductor manufacturing constraints. These requirements make magnetoelectric electronics not only a problem in condensed‐matter physics, but also a demanding challenge in materials engineering, device design, and system architecture.

Several comprehensive reviews [12, 13, 14, 15, 16] have already established the microscopic framework of multiferroicity, explaining how polar order and magnetic order can coexist through lone‐pair chemistry, geometric distortions, charge ordering, spin‐driven symmetry breaking, spin–lattice coupling, and interfacial effects. Building on this foundation, the present review focuses on how these materials principles translate into engineering requirements for beyond‐CMOS memory and logic. We first establish the physical basis and performance criteria for magnetoelectric switching. We then examine MeRAM, MESO, and emerging magnon‐mediated architectures and identify their device‐specific requirements. Guided by these requirements, we then examine magnetoelectric materials, focusing on single‐phase multiferroics, including type‐I, type‐II, and other single‐phase systems, and discuss how their structural and chemical features determine polarization, magnetic order, and magnetoelectric coupling. Finally, we consider fabrication, interface, circuit, and architectural constraints and outline priorities for future development.

## 2 Magnetoelectric Switching Principles and Evaluation Criteria

Magnetoelectric devices are promising candidates for beyond‐CMOS computing. Their distinction extends beyond switching energy: the two technologies encode information in fundamentally different physical variables. In a CMOS logic gate, the instantaneous logic state is represented by voltage, and hence charge, on a capacitive circuit node. Switching occurs when a gate voltage changes the electrostatic potential of a transistor channel, enabling carriers to charge or discharge that node. In a magnetic device, information is instead represented by the orientation of an ordered magnetic state. Switching therefore requires the magnetic system to cross, suppress, or reshape the free‐energy barrier separating stable configurations.

This distinction governs information retention. Conventional CMOS logic nodes are volatile: their electrical states are lost when the supply is removed or charge leaks away. This does not mean that all CMOS‐based storage is volatile, because nonvolatile memories can be integrated with CMOS, but nonvolatility is not intrinsic to the logic transistor. A magnetic state can remain stable without continuous power because magnetic anisotropy creates an energy barrier (∆E) between two orientations. Its retention is commonly characterized by the thermal‐stability factor (∆E/k_B_T). Magnetic nonvolatility consequently offers opportunities for normally‐off operation, embedded memory and computing architectures in which information storage is co‐located with logic.

The switching mechanisms are likewise different. A CMOS transistor uses gate voltage to modulate a transport barrier and thereby control channel current. Magnetic switching requires torque or an equivalent modification of the magnetic free‐energy landscape. Established approaches include magnetic‐field switching, spin‐transfer torque (STT), and spin‐orbit torque (SOT). STT and SOT deliver angular momentum through charge or spin currents and have enabled fast, durable devices compatible with magnetic tunnel‐junction readout. Their write operation, however, still entails current flow and associated resistive dissipation [17].

Magnetoelectric switching provides a different route. An applied electric field can modify magnetic anisotropy, exchange interactions, or interfacial coupling through polarization, strain, charge accumulation, or exchange‐mediated mechanisms [18, 19]. These effects can lower, tilt, or otherwise reconfigure the magnetic energy landscape and thereby assist or induce switching with little steady‐state write current. The relevant advantage is therefore not “charge‐free” operation: capacitive charging, leakage, peripheral circuitry and electrical readout still consume energy. Rather, magnetoelectric transduction can replace the large conductive write current required by conventional torque‐driven schemes (STT, SOT) with predominantly voltage‐driven control.

These advantages nevertheless involve important trade‐offs. A sufficiently large anisotropy barrier is needed for retention, whereas rapid, low‐voltage switching generally requires that this barrier be efficiently reduced or overcome. Magnetoelectric devices must therefore be assessed in terms of thermal stability, switching probability and latency, write voltage, endurance, readout margin and device variability. At the system level, transduction efficiency, interconnect capacitance, fan‐out, signal restoration and peripheral CMOS can dominate device‐level gains. Consequently, technological relevance must be established through benchmarking from the material and device levels through circuits and architectures, rather than from switching energy alone [20].

These criteria define the targets against which magnetoelectric technologies should be assessed; they do not by themselves establish readiness for direct CMOS replacement. Memory architectures primarily require low‐energy writing, non‐volatile retention and reliable readout, whereas logic additionally requires gain, fan‐out and signal restoration. The following section therefore examines each device family before returning to comparative benchmarking.

## 3 Beyond‐CMOS Memory and Logic Architectures Enabled by Magnetoelectric Coupling

Magnetoelectric coupling plays distinct functional roles across emerging beyond‐CMOS memory and logic architectures. Among these, MeRAMs exploit voltage‐controlled magnetic switching to enable non‐volatile information storage while avoiding the large charge currents and associated Joule heating inherent to conventional current‐driven architectures. In MESO logic, magnetoelectric switching provides the input‐side switch within an electrical‐to‐magnetic‐to‐electrical transduction chain, so output conversion and cascadability are equally important. Emerging magnon‐mediated devices instead use voltage‐controlled spin excitations for information transport. This section therefore separates the operating principles, experimental demonstrations, and key technological requirements of these architectures before comparing their performance and prospects for beyond‐CMOS information processing.

### 3.1 Magnetoelectric Random‐Access Memory (MeRAM)

Magnetoelectric random‐access memory (MeRAM) is a non‐volatile memory technology that enables voltage‐controlled magnetization switching by exploiting the inverse magnetoelectric effect, thereby avoiding the large charge currents and Joule heating intrinsic to conventional MRAM technologies. The basic memory cell in MeRAM is a voltage‐controlled magnetic tunnel junction (VC‐MTJ), which comprises a ferromagnetic reference layer with fixed magnetization, an ultrathin insulating tunnel barrier, and an ME‐coupled ferromagnetic free layer, as shown in Figure 2a below [21]. During the write operation, an applied voltage across the ME layer generates an electric field that modulates the magnetic anisotropy of the free layer, reducing the energy barrier between magnetic states and enabling low‐energy, non‐volatile magnetization switching without current‐driven spin torques [22]. The read operation is performed using the tunnelling magnetoresistance (TMR) effect [23], where the device resistance depends on the relative magnetization alignment of the free and reference layers, providing a robust and non‐destructive binary information readout.

**FIGURE 2:**
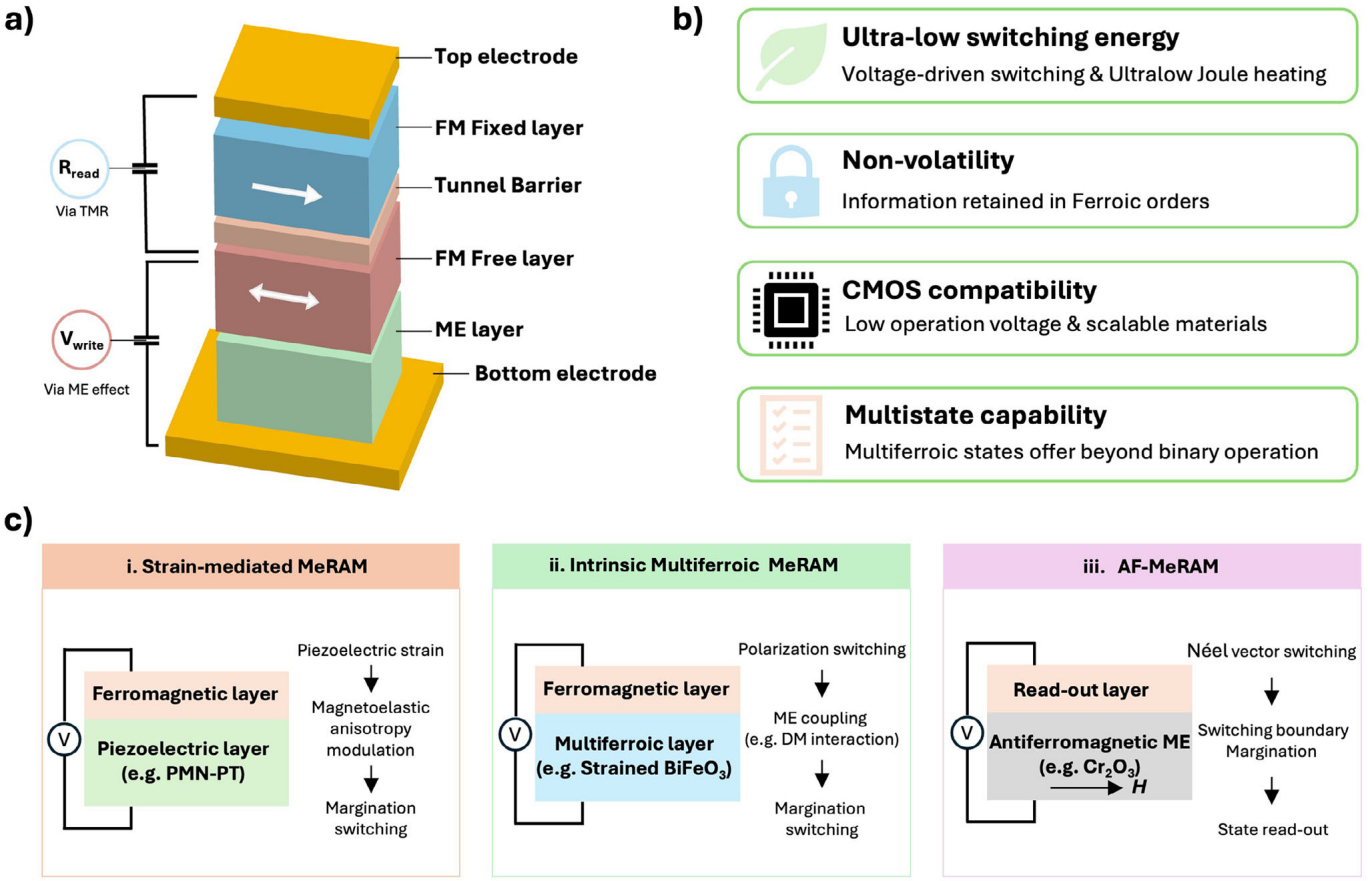
Magnetoelectric random‐access memory (MeRAM): device structure, core advantages, and representative prototype architectures. (a) Schematic illustration of a voltage‐controlled magnetic tunnel junction (VC‐MTJ)‐based MeRAM cell. (b) Key advantages of MeRAMs. (c) Representative prototype MeRAM architectures based on different inverse magnetoelectric mechanisms. (i) Strain‐mediated MeRAM (ii) Intrinsic multiferroic MeRAM (iii) Antiferromagnetic MeRAM (AF‐MeRAM).

Owing to its voltage‐driven operation, MeRAM offers several key advantages over existing and prototype non‐volatile memories, including ultralow write energy, high endurance, and CMOS‐compatible scalability (Figure 2b). Benchmarking and modelling studies have projected that optimized MeRAM architectures could achieve sub‐femtojoule‐level switching energy (<5 fJ/bit), compact cell size (20–30 F^2^), and outstanding endurance (>10^15^ cycles), positioning it close to the theoretical performance limits of non‐volatile memory technologies [22]. These characteristics make MeRAM a strong candidate for next‐generation memory systems aimed at overcoming the power‐density and scalability challenges of conventional CMOS‐based architectures.

To date, a variety of prototype MeRAM devices have been experimentally demonstrated based on different mechanisms of the inverse magnetoelectric effect (Figure 2c). One representative example is the strain‐mediated MeRAM reported by Hu et al., in which the magnetoelectric free layer consists of a piezoelectric PMN‐PT substrate coupled to a polycrystalline Ni magnetostrictive layer [24]. Upon application of a vertical electric field across the PMN‐PT, a large in‐plane piezoelectric strain is generated and elastically transferred to the Ni layer. The resulting strain‐induced magnetoelastic anisotropy drives a deterministic 90° in‐plane rotation of the magnetization. Phase‐field simulations further indicate that this strain‐mediated MeRAM concept offers substantial advantages over conventional MRAM, including an ultralow write energy of ∼0.16 fJ per bit, a projected storage density of ∼88 Gb in^−2^, and a sub‐10 ns switching time [24]. Beyond strain‐mediated approaches, intrinsic magnetoelectric coupling in single‐phase multiferroic materials has also been extensively explored for MeRAM applications. Early demonstrations using multiferroic antiferromagnets such as YMnO_3_ and LuMnO_3_ showed that electric‐field manipulation of the multiferroic order can modify interfacial exchange bias and induce reversible magnetization switching in an adjacent ferromagnetic layer [19, 25]. In 2014, Heron et al. demonstrated electric‐field‐induced magnetization reversal in strained BiFeO_3_ thin films, where ferroelectric polarization switching proceeds via two successive 71°/109° rotations. These polarization rotations are accompanied by corresponding rotations of the oxygen octahedra and the associated Dzyaloshinskii‐Moriya (DM) interaction vector, resulting in reversal of the weak ferromagnetic moment by 180° [5]. This mechanism yields an exceptionally large reverse magnetoelectric coefficient (α ∼ 1 × 10^−7^ s m^−1^), while the switching energy density (∼480 µJ cm^−2^) is more than an order of magnitude lower than that of current‐driven spin‐transfer torque devices [5]. This work established a route toward electrically written magnetic states in a room‐temperature single‐phase multiferroic, highlighting the potential of intrinsic magnetoelectric coupling for low‐power magnetic memory. More direct demonstrations of non‐volatile memory using single‐phase multiferroics were subsequently reported in room‐temperature hexaferrites. Zhai et al. demonstrated a non‐volatile memory based on the Z‐type hexaferrite Sr_3_Co_2_Fe_24_O_41_, in which binary information was encoded in the magnitude and sign of the first‐ and second‐order magnetoelectric coefficients, rather than in resistance [26]. In parallel, Kosub et al. demonstrated an antiferromagnetic MeRAM (AF‐MeRAM) based on the linear magnetoelectric effect of the antiferromagnet Cr_2_O_3_, achieving reliable, isothermal voltage‐controlled switching at room temperature [27]. In this system, electric‐field‐induced boundary magnetization reversal enables robust memory operation without the involvement of ferromagnetic domains. Owing to the absence of ferromagnetic hysteresis losses, the write threshold voltage is reduced by a factor of nearly 50 compared to ferromagnetic counterparts.

Beyond conventional binary MeRAM, multiferroic tunnel junctions (MFTJs) provide an additional route toward multilevel information storage by exploiting the coupled magnetic and ferroelectric order parameters. Unlike conventional MeRAM, in which information is primarily encoded in magnetic configurations and read through magnetoresistance, these MFTJs introduce an additional ferroelectric degree of freedom into the memory cell, enabling multiple resistance states through the coupled control of magnetic and ferroelectric order parameters. Gajek et al. demonstrated a proof‐of‐concept four‐state non‐volatile memory based on La_0.1_Bi_0.9_MnO_3_ (LBMO) multiferroic tunnel junctions, where the combination of tunnelling magnetoresistance (TMR) and tunnel electroresistance (TER) generated four distinguishable resistance states associated with different magnetic and ferroelectric configurations [28]. Subsequent studies demonstrated four‐state non‐volatile operation with reduced switching fields and larger resistance contrast, while later work further extended the concept to multilevel resistance states by exploiting intermediate ferroelectric polarization states [29, 30]. Thus, MFTJs expand the information space of conventional MeRAM by combining magnetic and ferroelectric states within a single tunnelling device, while retaining electrical resistance as the readout variable.

Furthermore, a fundamentally different approach goes beyond resistance‐based information storage by using the magnetoelectric response as the memory state. In this concept, rather than storing information in a magnetic configuration and detecting it through a resistance change as in conventional MeRAM, the magnetoelectric coefficient (α_ME_) is directly used as the information‐carrying variable. Shen et al. demonstrated this concept in a PMN‐PT/Terfenol‐D multiferroic heterostructure, where the magnitude and sign of α_ME_ were controlled through the relative ferroic configurations and, in particular, by electrically tuning the fraction of oppositely polarized ferroelectric domains, which enabled stable up to 8‐level non‐volatile memory states at room temperature [31]. The write operation is induced by an electric‐field pulse, similar to ferroelectric memory, whereas readout is fundamentally different: a small AC magnetic field is applied to generate a magnetoelectric voltage, and the resulting response is used to identify the stored α_ME_ state. This non‐destructive readout can further be implemented using a shared reading coil for multiple memory cells. Subsequent studies extended α_ME_ ‐based memory to nonlinear magnetoelectric devices and flexible organic memristors, demonstrating multilevel operation and, in the latter case, the use of the magnetoelectric coefficient as a synaptic weight for neuromorphic applications [32, 33].

### 3.2 Benchmark Of Established Memories and MeRAM Routes

Table 1 compares strain‐mediated, intrinsic‐multiferroic, and antiferromagnetic MeRAM with established FeRAM and STT‐MRAM and with projected VCMA‐MeRAM. FeRAM offers voltage‐controlled switching but requires destructive read–restore operation, whereas STT‐MRAM provides magnetic storage and nondestructive TMR readout but relies on current‐driven writing. MeRAM aims to combine the advantages of both: voltage‐controlled writing, magnetic nonvolatility, and nondestructive electrical readout.

**TABLE 1:** Representative numerical benchmark of established memories and MeRAM routes.

Metric	Established memories		Projected reference	Experimental MeRAM routes		
	FeRAM (C/A/R) [34, 35, 36, 37]	STT‐MRAM (C/R) [38]	VCMA‐MeRAM (2018 P) [22]	Strain‐mediatedMeRAM (E) [39]	Intrinsic‐multiferroic MeRAM (E) [10]	Antiferromagnetic MeRAM (E) [40]
Cell area or device scale	1T1C PZT/HZO 6–30 F^2^	1T1MTJ 6–30 F^2^	V‐MTJ 20–30 F^2^ (P)	pMTJ/PMN–PT 10 µm / 0.2 mm	CoFe/BFO/Pt 500 150 nm^2^ / 30 nm	Pt/Cr_2_O_3_/Ir 5 × 25 µm^2^ / 15 nm
Capacity, density, or array	8 Mb (2021 R) 16 Mb (current C)	1 Gb (2021 R)	2 Gb cm^−^^2^ (P)	1 device	1 device	1 device 100 nm patterned bits^a^
Qualified retention	≥10 years at 85 °C (C)	>10 years (R)	NR	NR	NR	NR
Endurance	≈10^1^ ^5^ accesses (C/R) >10^1^ ^2^ cycles (A)	≈10^1^ ^5^ cycles (R)	≈10^1^ ^5^ cycles (P)	NR	NR	NR
Read time	10–100 ns (R) 5 ns (A)	≈10 ns (R)	1–5 ns (P)	NR	NR	NR
Write time or applied pulse	10–100 ns (R) 7 ns (A)	≈10 ns (R)	<1 ns (P)	NR	200 µs pulse	NR 100 µs pulse^*^
Cell energy	≈0.1 pJ (R) 0.7 pJ bit^−^^1^ at 2 V (A)	≈0.1 pJ (R)	Read: 1–5 fJ (P) Write: <5 fJ (P)	NR	NR	NR
Write drive and condition	1.3–2.0 V (HZO arrays)	Current through MTJ	Voltage / VCMA	±10 kV cm^−^^1^ ≈±200 V; H = 0	±2 V 300 K; H = 0	<0.8 V 275 K; μ_0_H ≈ 0.5 T
Electrical readout	Charge DR + restore	TMR NDR	TMR NDR	TMR = 138.9% ΔR/R = 37.8%; NDR	ΔV ≈ 1 µV at 100 µA NDR	AHE at 100 µA NDR
Integration status	Commercial / integrated	Commercial / embedded	Simulated array	Discrete device	Discrete device	Patterned, not independently addressed^*^

**Notes**. C, commercial; A, integrated array; E, experimental device; R, 2021 commercial‐memory benchmark reported in 2022; P, 2018 VCMA‐MeRAM array‐level projection; NR, not reported; DR, destructive read; NDR, nondestructive read in operating principle. Pulse width is not a time‐resolved switching latency. Values within a column may originate from separate reports and are not necessarily co‐demonstrated. The VCMA‐MeRAM column is a reference target and must not be assigned to the three multiferroic MeRAM routes.

^a^ The 100‐nm Cr_2_O_3_ patterned bits and the field‐free −11‐V, 100‐µs B:Cr_2_O_3_ result were obtained in separate experiments and were not co‐demonstrated with the <0.8‐V electrical device.

FeRAM and STT‐MRAM remain the most relevant benchmarks, with representative cell areas of 6–30 (F^2^), retention exceeding ten years, endurance approaching (10^15^) cycles, and energies near 0.1 pJ per cell [38]. STT‐MRAM offers approximately 10‐ns read and write times, while FeRAM has reached 5‐ns read and 7‐ns write times in hafnia‐based arrays. The VCMA‐MeRAM values in Table 1 are projections rather than experimental results; they predict sub‐nanosecond writing, 1–5‐ns readout, and energies below approximately 5 fJ bit(^−1^) [22].

The three experimental MeRAM routes offer distinct advantages. Strain‐mediated MeRAM provides the largest demonstrated electrical read contrast, including a 138.9% TMR ratio, but current demonstrations rely on bulk piezoelectric substrates, high voltages, and non‐deterministic magnetic reconfiguration [39]. Intrinsic‐multiferroic MeRAM enables direct voltage‐to‐magnetism conversion without a separate transducer and has demonstrated electrical write–read operation, but its read signal is small and switching reproducibility remains limited [10]. Antiferromagnetic MeRAM offers negligible stray fields, weak intercell interaction, and potential high‐speed operation; however, low‐voltage switching, field‐free operation, and electrical readout have so far been demonstrated separately [40, 41].

Overall, multiferroic MeRAM has not yet surpassed FeRAM or STT‐MRAM in measured speed, energy, endurance, retention, or density. Its main promise is the combination of electric‐field writing, magnetic storage, and nondestructive readout. Practical progress will require deterministic binary switching, adequate read margin, low write‐error rates, long retention, high endurance, and independently addressable arrays.

### 3.3 Magnetoelectric Spin‐Orbit‐Coupled (MESO) Logic Devices

#### 3.3.1 Device Architecture and Signal Pathway

Beyond MeRAM, the magnetoelectric (ME) effect has also been extended to magnetoelectric spin‐orbit (MESO) logic devices. As a representative beyond‐CMOS logic paradigm rooted in quantum nanoelectronics and nano‐magnetism, MESO logic combines electric‐field control of magnetic states with spin‐orbit‐mediated charge‐spin interconversion [42]. This operating principle offers a compelling route toward logic devices with markedly improved energy efficiency, high switching speed, and intrinsic non‐volatility, making MESO a promising candidate for future energy‐efficient computing architectures.

The concept of MESO logic was originally proposed by a collaborative team from Intel, the University of California, Berkeley, and Lawrence Berkeley National Laboratory [8]. By exploiting the coupled dynamics among electric‐field control, magnetic order parameter rotation, and strong spin‐orbit coupling (SOC), MESO devices enable energy‐efficient and non‐volatile logic operation. A schematic illustration of a typical MESO unit structure is shown in Figure 3a. In the write process, an input voltage applied across the magnetoelectric (ME) layer generates an electric field that switches the magnetization of the adjacent ferromagnetic layer through ME coupling (Figure 3b). During signal propagation and readout, the magnetic state switching generates a spin‐polarized current, which is subsequently injected into a spin‐orbit coupling (SOC) layer and converted into an electrical output through spin‐charge conversion mechanisms such as the inverse spin Hall effect (ISHE) and inverse Rashba‐Edelstein effect (IREE) (Figure 3c) [43]. This output electrical signal serves directly as the input for the subsequent MESO logic unit, thereby enabling cascaded logic operations without the need for intermediate charge storage [8, 44].

**FIGURE 3:**
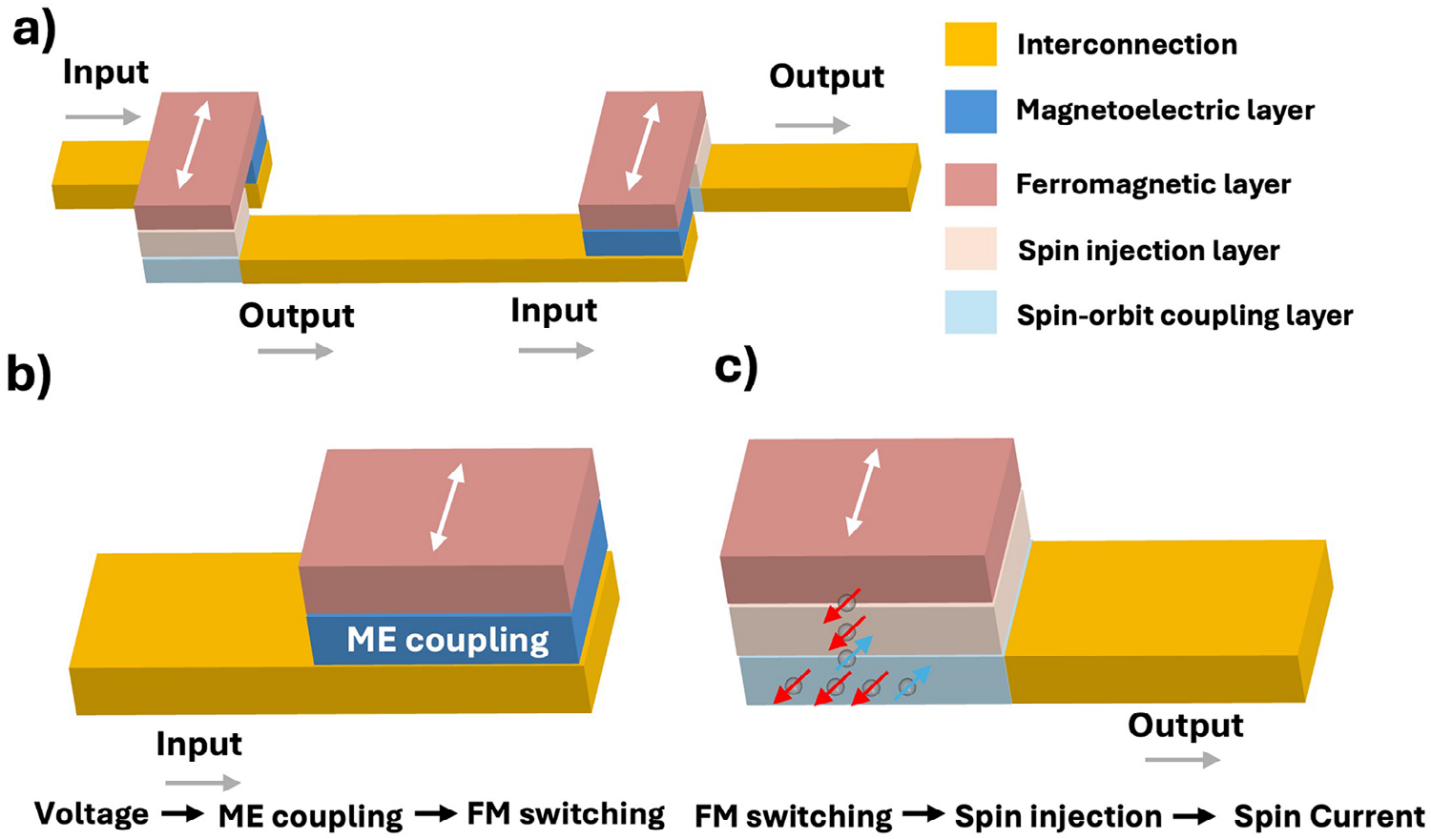
Magnetoelectric spin‐orbit (MESO) logic: operating principle, device components, and current optimization strategies. (a) Schematic illustration of the overall MESO logic architecture and signal propagation pathway. (b) Input switching unit of the MESO device. The applied electric field across the ME layer modulates the magnetic anisotropy or exchange interaction at the FM/ME interface, enabling voltage‐driven magnetization switching without current‐induced spin torques. (c) Output spin‐charge conversion unit. The spin current generated by the FM layer is injected into the SOC material, where spin‐orbit interactions convert the spin signal into a measurable electrical output. (Red and blue arrows indicate the up and down spins injected from the ferromagnetic layer.) [Graphics are adapted from reference [8]]

#### 3.3.2 Input Switching and Voltage Scaling

At present, achieving attojoule‐level energy consumption in MESO devices is primarily limited by two key bottlenecks. First, the driving voltage of the ME switching element must be reduced from the present level of several volts to the sub‐100 mV regime [42]. Second, the output voltage generated by the spin‐orbit coupling element must be increased from the hundreds of microvolts to the hundreds‐of‐millivolts range [45]. Reducing the ME switching voltage critically requires a detailed understanding of polarization and magnetization switching dynamics in ultrathin multiferroic films. In particular, the switching voltage generally scales with film thickness, and meanwhile ferroelectric switching in BiFeO_3_ thin films is widely understood to proceed via nucleation and growth of reversed polarization domains. This behavior is well described by the Kay‐Dunn model, in which the coercive field scales with film thickness to the power of −2/3 [46]. Recently, a notable breakthrough was achieved by introducing La‐doped BiFeO_3_ ultra‐thin films, which significantly reduced the coercive field at room temperature [47, 48]. As a result, the exchange‐coupling‐mediated magnetization reversal voltage has been demonstrated to be as low as 150 mV for the 6 nm La‐BiFeO_3_ film—approaching the theoretical voltage threshold required for attojoule‐scale MESO operation [47]. Another important strategy is improving the efficiency of voltage‐controlled magnetic switching through interface engineering. In BiFeO_3_‐based heterostructures, enhanced interfacial magnetoelectric coupling can strengthen polarization‐mediated magnetization switching and reduce the operating voltage required for magnetic reversal [49, 50]. Figure 4a summarizes representative voltage‐scaling pathways for MESO‐relevant magnetoelectric switching mechanisms, highlighting that recent interface‐coupled ultrathin multiferroic heterostructures are approaching the sub‐100 mV regime required for attojoule‐scale MESO operation.

**FIGURE 4:**
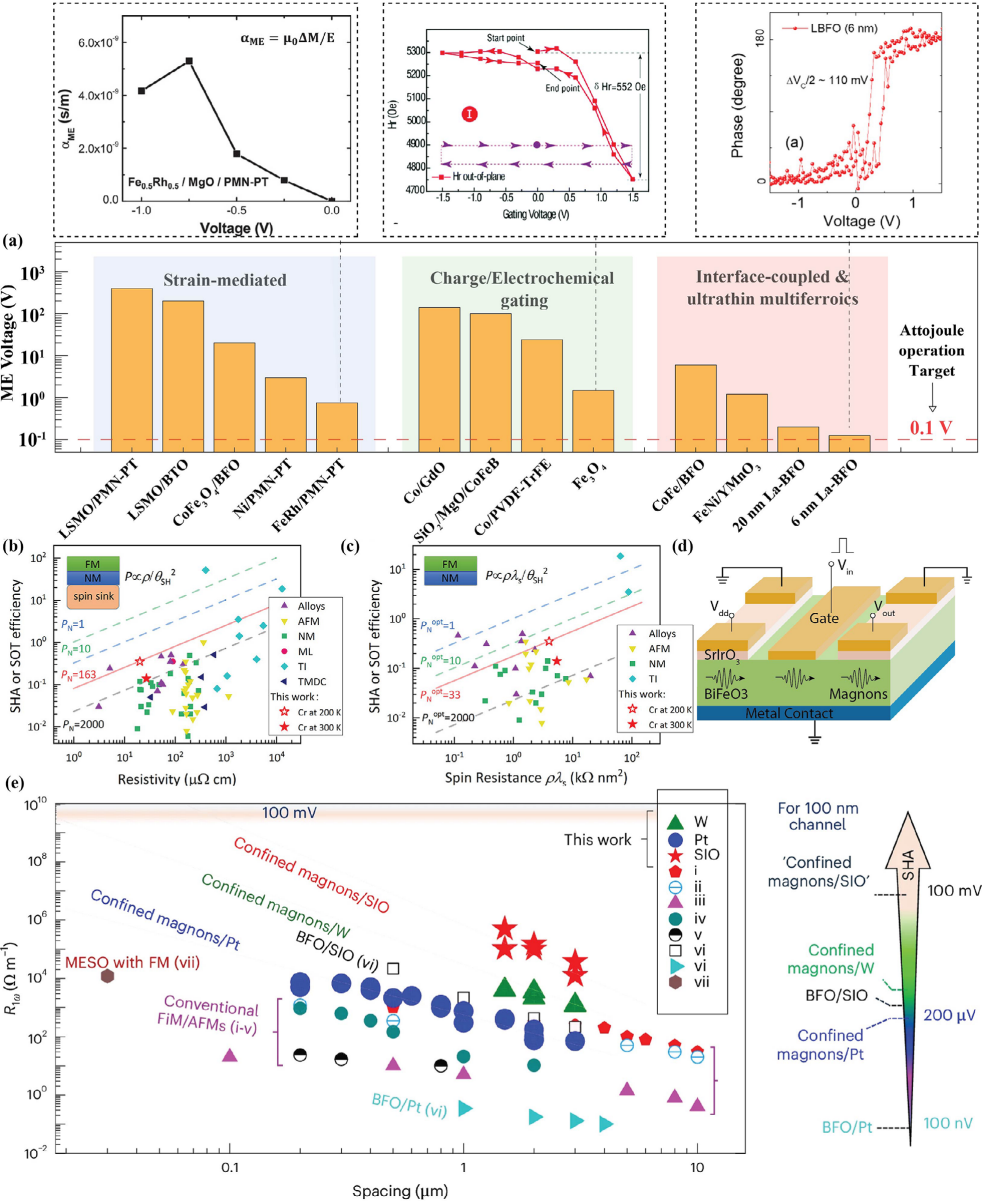
Current development roadmap of magnetoelectric spin‐orbit (MESO) logic devices. (a) Representative operating voltages reported for different magnetoelectric switching mechanisms relevant to the sub‐100 mV voltage target required for attojoule‐class MESO operation [5, 19, 47, 48, 58, 59, 60, 61, 62, 63, 64, 65, 66]. Summary of (b) resistivity and (c) normalized power consumption as a function of SOT efficiencies for a series of representative material systems. (Reprinted from [56]) (d) Schematic illustration of an emerging ferromagnetic‐free MESO architecture based on voltage‐controlled confined magnon transport in antiferromagnetic oxide heterostructures. (Reprinted from [42]) (e) Spin‐Hall resistance calculated from the ISHE voltage and scaled with the length of the wire as a function of the SO metal spacing. This shows the benchmarking of the output voltage measured in non‐local spin transport in the range of material systems. (Reprinted from [67]).

#### 3.3.3 Output Conversion and Cascadability

In parallel, substantial efforts have been devoted to increasing the MESO output voltage by improving spin‐to‐charge conversion efficiency in the spin‐orbit coupling (SOC) layer [51]. One major strategy involves the development of advanced SOC materials, including heavy metals with large spin Hall angles [52], topological insulators (TIs) [53, 54], and oxide heterostructures [55]. These materials exhibit strong spin‐orbit interactions and enhanced spin‐charge conversion efficiency, while topological systems additionally benefit from spin‐momentum locking, enabling more effective conversion of spin signals into electrical output voltages. Figure 4b,c, adapted from the road map of SOC materials by Shao et al., illustrate the relationship among SOT efficiency, resistivity, and power consumption for representative SOC material systems relevant to MESO output optimization [56]. However, many of these emerging quantum materials still face challenges in CMOS compatibility and large‐scale integration. In addition to material optimization, structural amplification strategies have also been explored. For example, integrating an additional magnetic tunnel junction (MTJ) into the MESO architecture can enhance the injected tunnelling current and increase the output voltage to the millivolt range [57]. Nevertheless, the additional driving voltage required for MTJ operation may introduce extra energy overhead and circuit complexity.

Despite substantial progress in both ME switching and SOC material optimization, the output voltage of conventional ferromagnet‐based MESO architectures remains limited by the relatively low efficiency of spin transport and spin‐to‐charge conversion in conventional ferromagnetic architectures.

#### 3.3.4 Antiferromagnetic and Magnon‐Mediated Devices

Multiferroic antiferromagnets, particularly BiFeO_3_ (BFO), offer an alternative route by enabling electrical control of antiferromagnetic spin dynamics and magnon transport, thereby opening new possibilities for MESO architectures based on antiferromagnetic magnon transport and magnon‐mediated signal propagation [68]. Such architectures are generally referred to as antiferromagnetic magnetoelectric spin‐orbit (AF‐MESO) architectures. In this device structure (Figure 4d), the magnetoelectric layer serves as the magnon‐transport channel, with magnons acting as the signal carriers. The magnetoelectric layer is sandwiched between the source and drain electrodes, while a spin‐orbit metal electrode is integrated at the source interface. A magnon flux is generated in the magnetoelectric layer through the spin Hall effect (SHE) at the source interface. This non‐equilibrium magnon flux transports angular momentum toward the drain, where it is converted into a voltage output through the ISHE in the nonlocal geometry. The gate voltage controls the ferroelectric polarization of the magnetoelectric layer, thereby modulating the efficiency of magnon transmission and producing distinct output‐voltage states [42].

BFO simultaneously hosts ferroelectric and antiferromagnetic orders at room temperature, with strong coupling between these order parameters. Early studies established the electrical control of antiferromagnetic order in BFO [69, 70]. An important subsequent advance was reported by Rovillain et al., who demonstrated direct electric‐field control of spin‐wave modes in room‐temperature BFO [71]. Spin‐wave frequencies above 600 GHz could be tuned by more than 30% in a non‐volatile manner, demonstrating that the magnetoelectric coupling in BFO can be exploited not only to manipulate static antiferromagnetic states but also to electrically control high‐frequency spin dynamics [71]. Subsequent studies progressively extended this concept from controlling spin‐wave modes to engineering and manipulating magnon propagation through the underlying antiferromagnetic spin texture. Strain and electric‐field engineering were shown to stabilize and reconfigure different antiferromagnetic textures and spin‐cycloid states in BFO, while epitaxial anisotropy and ferroelectric polarization can further determine the propagation direction of the spin cycloid [72]. Chemical and domain engineering have also enabled single‐domain BFO with a stable spin‐cycloid variant, leading to strongly anisotropic magnon transport [73]. In parallel, deliberate engineering of the underlying spin‐texture lattice enabled an epitaxial magnonic crystal with pronounced anisotropic spin transport, providing a more general strategy for controlling magnon propagation in multiferroic antiferromagnets [74]. More recently, reversal of the ferroelectric polarization was shown to modulate chiral magnon transport in BFO at room temperature, with the resulting magnon spin torque capable of switching adjacent magnetic layers and enabling an all‐oxide logic architecture [75]. These advances have increasingly shifted multiferroic magnonics from fundamental control of spin excitations toward functional information processing. For example, Chai et al. demonstrated voltage‐controlled multiferroic magnon torque in BFO heterostructures, where ferroelectric polarization modulates the non‐collinear antiferromagnetic structure and consequently the magnon‐mediated spin–orbit torque acting on an adjacent ferromagnet. By exploiting the coupled non‐volatile ferroelectric and magnetic states, reconfigurable logic‐in‐memory operations were demonstrated in a single device [76]. A remaining challenge, however, is to efficiently confine and transmit magnons over nanoscale distances while generating a sufficiently large electrical output for practical logic integration. Recently, Husain et al. demonstrated magnon confinement in an epitaxial LaFeO_3_/BiFeO_3_/LaFeO_3_ all‐antiferromagnetic heterostructure, where magnons are confined within an ultrathin BFO antiferromagnetic layer. This confinement enhanced the output voltage generated by magnon transport by several orders of magnitude [67]. As benchmarked in Figure 4e, reproduced from Husain et al. [67], the confined‐magnon architecture exhibits substantially enhanced spin‐Hall resistance compared with many reported ferrimagnetic and antiferromagnetic systems. Importantly, the output can be further enhanced by employing spin‐orbit materials with higher spin–charge conversion efficiency, like epitaxial SrIrO_3_. Together with further device scaling, the combination of magnon confinement and efficient spin‐charge conversion provides a potential pathway toward the ∼100 mV output targeted for MESO operation.

These developments suggest that future MESO platforms may gradually evolve from conventional ferromagnetic spin‐current architectures toward magnonic and anti‐ferromagnetic spin‐logic architectures.

### 3.4 Comparative Benchmarking of Established CMOS (FET) and MESO Technologies

Since MESO was proposed in 2018 [8], research in magnetoelectric logic has progressively shifted from conceptual device proposals to experimentally grounded, interface‐driven magnetoelectric systems, with voltage‐controlled magnetic anisotropy emerging as a leading pathway toward practical low‐energy switching. Projected device‐level analyses suggest that MESO‐like logic could reach switching energies in the 10–100 aJ range, corresponding to an estimated 10–30‐fold reduction relative to CMOS under comparable assumptions. Despite this progress, large‐scale logic integration remains unresolved [8]. As such, evaluating the viability of magnetoelectric logic requires benchmarking the technology at the circuit and architectural levels, rather than relying on isolated device metrics.

Benchmarking magnetoelectric logic requires projected device metrics to be distinguished from experimentally demonstrated performance. As shown in Table 2, projected ME oxide architectures suggest low‐voltage operation near 0.1 V and switching energies approaching ∼10^−^^1^ ^7^ J, placing them in an attractive regime for non‐volatile, low‐energy logic. By contrast, many experimental devices still require substantially higher switching voltages, typically ∼1.8–2.0 V. In a practical logic circuit, however, a device cannot be assessed only as an isolated switch. The output of one gate must serve as the input to the next gate, often to several gates at once, while preserving a clear distinction between logical “0” and “1”. If the readout voltage generated by one ME stage is smaller than the voltage required to switch the next, the signal cannot propagate reliably through the circuit. This requirement imposes constraints on voltage gain, signal restoration, and fan‐out. As shown in Figure 5a, the normalized benchmarking suggests that magnetoelectric (ME) logic oxides offer a distinct performance profile relative to CMOS field‐effect transistors (FETs), with particularly favorable behavior in switching voltage, based on the predicted value, where lower values are preferred. In contrast, FETs appear to retain advantages in metrics such as density and switch speed, indicating that they remain stronger candidates for high‐integration, high‐speed logic applications. These trade‐offs demonstrate that individual device metrics alone are insufficient to assess the potential of magnetoelectric computing, motivating the need for systematic benchmarking across multiple levels of abstraction.

**TABLE 2:** Benchmarking magnetoelectric logic against CMOS (FET). Values represent a combination of projected limits and experimental demonstrations. The limited output voltage in magnetoelectric readout prevents gain and fan‐out, restricting circuit‐level operation. (Data from IRDS 2024 [1] and recent works [11, 75]).

Metric	Direction	FET projected	FET demonstrated	ME logic oxides projected	ME logic oxides demonstrated
Cell size	lower is better	50 nm	56 nm	50–100 nm	∼100–200 nm
Density	higher is better	1.0 × 10^10^ cm^−^^2^	1.08 × 10^10^ cm^−^^2^	∼1.0 × 10^10^ cm^−^^2^	not scalable yet (device‐level only)
Switch speed	higher is better	12 THz (intrinsic)	0.4 THz	∼100 GHz	ps regime (∼GHz equivalent)
Circuit speed	higher is better	61 GHz	∼10 GHz	∼50 GHz	not demonstrated (fan‐out limit)
Switch energy	lower is better	3.0 × 10^−^^1^ ^8^ J	1.1 × 10^−^^1^ ^6^ J	∼10^−^^1^ ^7^ J	>10^−^^1^ ^7^ J (realistic, not yet optimized)
Circuit energy	lower is better	1.2 × 10^−^^1^ ^7^ J	1.0 × 10^−^^1^ ^5^ J	∼10^−^^1^ ^6^–10^−^^1^ ^5^ J	unknown (no circuits)
Subthreshold slope	lower is better	60 mV/dec	62 mV/dec	N/A	N/A
Readout voltage	higher is better	∼0.5–0.7 V	∼0.5 V	∼0.1 V (target)	1 µV
Required switching voltage	lower is better	∼0.5 V	∼0.7 V	∼0.1 V	±1.8–2.0 V
Gain / fan‐out	required	**✓**	**✓**	unclear	✕ not satisfied (critical bottleneck)

**FIGURE 5:**
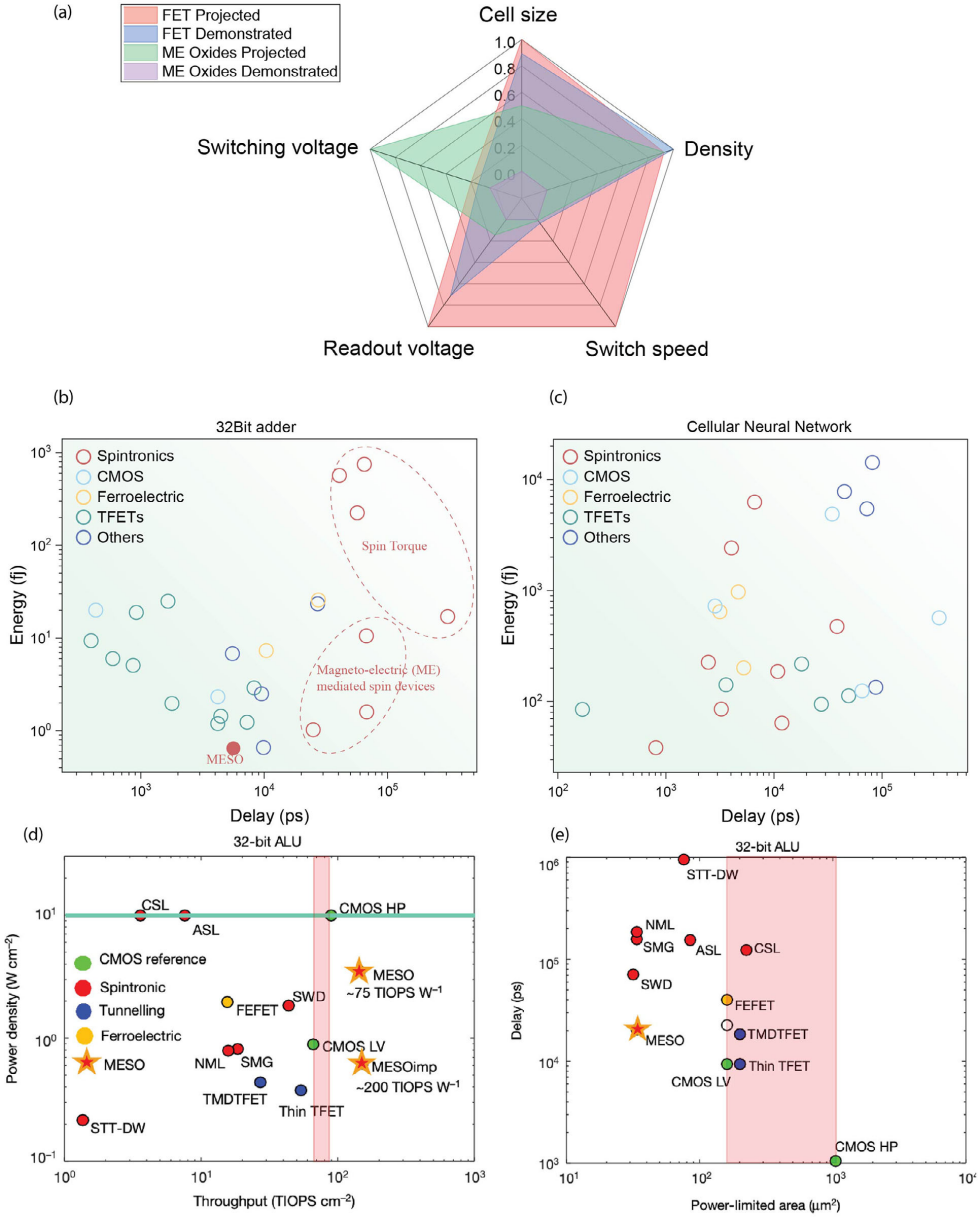
Benchmarking of beyond‐CMOS devices. (a) Normalized performance benchmarking of Magnetoelectric (ME) Logic Oxides vs. CMOS Field‐Effect Transistors (FETs). (b, c) System‐level benchmarking of beyond‐CMOS devices for logic and neuromorphic workloads. Data are compiled from Refs [7, 11, 77]. (d) Comparison of the computational throughput of 32‐bit arithmetic logic units (ALUs) as a function of power density for CMOS and representative beyond‐CMOS technologies. Throughput is expressed in tera‐integer operations per second per unit area (TIOPS cm^−^^2^). A maximum power density of 10 W cm^−^^2^ is imposed to ensure a fair comparison across different device technologies. (e) Comparison of the computational delay of 32‐bit ALUs as a function of the power‐limited area for CMOS and representative beyond‐CMOS technologies, using the same maximum power‐density constraint of 10 W cm^−^^2^. Reprinted with permission from Ref. [8]. Copyright 2019 Springer Nature.

Building on the benchmarking framework established by Nikonov and Young [6], subsequent studies [7] evaluated magnetoelectric devices within representative computing workloads. Rather than comparing isolated switching elements, these analyses assessed complete logic circuits under consistent assumptions, providing a more realistic measure of computational performance. As shown in Figure 5b, voltage‐controlled spintronic devices, including ME‐MTJ, CoMET, and spin‐wave devices, consistently achieve lower switching energy than current‐driven spintronic technologies by eliminating the Joule heating associated with spin‐transfer torque. Nevertheless, their operating speed remains limited by nanosecond‐scale magnetization reversal and domain‐wall propagation, whereas advanced CMOS continues to operate in the picosecond regime despite its higher operating voltage and static leakage. Interestingly, this performance gap narrows considerably for Cellular Neural Network workloads (Figure 5c), indicating that the principal advantage of magnetoelectric devices lies not in replacing CMOS gate‐for‐gate, but in architectures that exploit non‐volatility, majority logic and reduced data movement.

The emergence of magnetoelectric spin–orbit (MESO) logic represents an important progression from device‐level optimization to architecture‐level benchmarking. Rather than evaluating magnetoelectric switching solely as an energy‐efficient alternative to spin‐transfer torque, MESO benchmarking assessed the system‐level implications of integrating magnetoelectric devices into complete logic architectures. As shown in Figure 5d, MESO achieves substantially higher computational throughput under a given power‐density constraint than earlier spintronic logic concepts while approaching the efficiency of low‐voltage CMOS. Similarly, Figure 5e places MESO in a favorable region of the delay‐area design space, highlighting its potential to combine competitive computational performance with high logic density. These results suggest that the benefits of magnetoelectric technologies extend beyond reducing switching energy, enabling improvements at the architecture level through the combination of low‐voltage operation, compact logic implementation, and non‐volatility. The underlying device structure and operating principles of MESO are discussed in detail in Section 2.

Despite the encouraging benchmark results, comparisons with CMOS should be interpreted within the broader technology landscape. The International Roadmap for Devices and Systems (IRDS) [1] continues to regard most beyond‐CMOS logic concepts as long‐term research technologies, while placing greater confidence in CMOS extension because of its mature fabrication processes, interconnect infrastructure, and highly optimized design ecosystem. Consequently, the objective of magnetoelectric logic is unlikely to be a one‐to‐one replacement of CMOS. Instead, its greatest potential lies in application domains where the unique attributes of voltage‐controlled switching, intrinsic non‐volatility, and reduced standby power outweigh the additional complexity associated with materials integration. Examples include logic‐in‐memory, compute‐near‐memory and intermittently powered edge systems, where minimizing data movement and eliminating standby energy are more important than maximizing switching speed. From this perspective, future benchmarking should increasingly evaluate application‐specific system performance rather than relying solely on comparisons with conventional CMOS logic.

## 4 Magnetoelectric Materials

The development of ultralow‐power magnetoelectric logic and memory technologies requires materials that combine strong electric‐field control of magnetic order with reliable switching, stable magnetic states, and compatibility with scalable device integration.

The device relevance of a magnetoelectric material depends not only on the coexistence of ferroelectric and magnetic orders, but also on the strength, controllability, and reversibility of their coupling under device‐relevant conditions. For beyond‐CMOS applications, key considerations include room‐temperature operation, low‐voltage electric‐field control of magnetic order, deterministic switching, magnetic‐state stability, spin dynamics, and compatibility with thin‐film processing. These priorities vary across device architectures. MeRAM requires a stable non‐volatile magnetic state that can be reversibly controlled by a low‐voltage electric field, whereas MESO additionally requires the efficient generation of spin signals through magnetoelectric switching, followed by effective spin‐to‐charge conversion. Emerging magnon‐based architectures place greater emphasis on antiferromagnetic spin dynamics and the generation, propagation, and detection of magnon signals. The relevance of each material class is therefore assessed according to the microscopic origin of its magnetoelectric coupling and its ability to support voltage‐controlled switching, magnetic‐state retention, spin‐signal transduction, and magnon‐mediated information transport.

Multiferroics are materials that exhibit two or more primary ferroic order parameters within a single material system and constitute a key materials platform for magnetoelectric (ME) devices. Depending on how these ferroic orders arise, multiferroics are broadly classified into single‐phase and composite systems. In single‐phase multiferroics, multiple ferroic orders coexist intrinsically within a single crystallographic phase, giving rise to built‐in magnetoelectric coupling. By contrast, composite multiferroics are artificially engineered heterostructures composed of two or more distinct phases, typically a ferroelectric phase and a magnetic phase, in which the effective multiferroic response is mediated by interfacial coupling between them.

The intrinsic coupling in single‐phase multiferroics enables direct electric‐field control of magnetization (and vice versa) without the need for mechanical intermediaries. This direct coupling can, in principle, yield lower energy consumption and faster switching dynamics than those achievable in composite systems. In addition, the absence of internal interfaces eliminates many of the reliability issues commonly encountered in composites, such as interfacial chemical interdiffusion, fatigue, and aging. Furthermore, because a single‐phase multiferroic requires only one functional layer whose performance does not rely on strain transfer across interfaces, it is inherently more amenable to device miniaturization, high‐density integration, and reduced fabrication complexity in integrated circuits.

Despite these attractive advantages, the technological deployment of single‐phase multiferroics remains severely constrained by the fact that most known compounds exhibit robust multiferroic behavior only at cryogenic temperatures, rendering them unsuitable for mainstream commercial applications. In this section, we will discuss some single‐phase multiferroics that retain functional multiferroic properties at or near ambient temperature.

Single‐phase multiferroics are commonly divided into type‐I and type‐II classes. Type‐II multiferroics, where ferroelectricity is generated in specific magnetic ordering, are sometimes called magnetism‐driven multiferroics. All other single‐phase multiferroics that do not have a magnetic origin of ferroelectricity can be categorized as type‐I multiferroics.

### 4.1 Type‐I Multiferroics

Bismuth ferrite (BiFeO_3_, BFO) occupies a unique position as one of the very few single‐phase compounds that simultaneously exhibit long‐range ferroelectric and magnetic order well above room temperature. Its ferroelectric Curie temperature (*T*_C_ = 1100 K) and antiferromagnetic Neel temperature (*T*_N_ = 640 K) ensure that both ferroic orders are intrinsically stable under ambient conditions, distinguishing BiFeO_3_ from most single‐phase multiferroics whose functionality is limited to low temperatures [78].

Early BFO ceramic samples typically exhibited low apparent polarization values (<10 µC cm^−2^), largely due to high leakage currents [78]. Subsequent improvements in crystal growth enabled the observation of spontaneous polarization values of 90 µC cm^−2^ at 300 K [79]. Remanent polarization values of 110 µC cm^−2^ at room temperature have further been reported for BFO films grown on lattice‐matched substrates such as SrTiO_3_ and DyScO_3_ [79]. The coercive electric field typically lies between 50 and 150 kV cm^−1^ for bulk samples and increases to 200–500 kV cm^−1^ in thin films [78, 79].

BFO is a G‐type antiferromagnet with weak spin canting. In bulk form, the long‐wavelength cycloidal spin structure effectively cancels macroscopic magnetization, resulting in room‐temperature magnetization values typically below 0.05 emu g^−1^ [78]. In epitaxial thin films, strain‐induced suppression of the cycloidal spin modulation leads to a room‐temperature magnetization of ∼0.1 μ_B_ per Fe ion [80]. These values enable observable magnetic hysteresis and exchange coupling with adjacent ferromagnetic layers, including room‐temperature exchange bias fields on the order of 50–200 Oe in BFO‐based heterostructures [80]. Chemical substitution provides an effective route to enhancing magnetic performance. Rare‐earth‐doped BFO systems (e.g., La‐, Nd‐, and Sm‐doped) commonly exhibit room‐temperature magnetization values in the range of 0.1–1.5 emu g^−1^, representing an order‐of‐magnitude enhancement compared with undoped bulk BFO [78]. Importantly, these enhancements are achieved while maintaining magnetic ordering temperatures well above 300 K [81].

The intrinsic linear ME coupling in pristine bulk BFO is relatively weak. Room‐temperature linear ME coefficients are typically below 1 mV cm^−1^ Oe^−1^, reflecting the small net magnetization and leakage‐related measurement challenges [78]. In epitaxial thin films, a ME coupling coefficient on the order of ten V cm^−1^ Oe^−1^ has been demonstrated at room temperature, attributed to the non‐volatile 90° and 180° magnetization switching [80, 82]. Chemical substitution and solid‐solution formation can further enhance magnetoelectric coupling. Rare‐earth‐doped BFO compositions typically exhibit linear ME coefficients of 20 mV cm^−1^ Oe^−1^ at 300 K, particularly near structural phase boundaries, while BFO‐based solid solutions engineered to exhibit multiferroic phase boundaries have demonstrated even larger room‐temperature ME coefficients, reaching 50 mV cm^−1^ Oe^−1^ (Figure 6a) [81].

**FIGURE 6:**
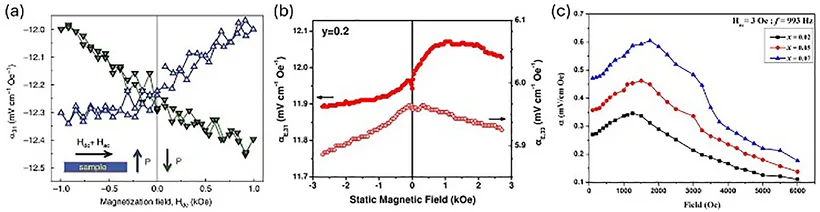
Representative magnetoelectric response characteristics of selected multiferroic materials. (a) Magnetoelectric voltage coefficient as a function of a static magnetization field for Bi_0.68_Pb_0.32_Fe_0.655_Mn_0.025_Ti_0.32_O_3_ ceramics. The measuring configuration is illustrated in the inset [81]. (b) Magnetoelectric voltage coefficient as a function of the bias magnetic field in longitudinal and transverse modes at 1 kHz and room temperature for Pb(Fe_0.5_Ta_0.5_)_0.2_(Zr_0.53_Ti_0.47_)_0.8_O_3_ [85]. (c) Variation of the magnetoelectric coupling coefficient with dc bias field for Bi_4‐x_Sm_x_Ti_3‐x_Ni_x_O_12_, with x = 0.02, 0.05, 0.07 [91].

Pb‐based perovskite oxides constitute another extensively investigated class of single‐phase multiferroic materials that operate at or near room temperature. In contrast to BFO, where multiferroicity is intrinsic to a single compound, Pb‐based multiferroics are largely achieved through solid‐solution engineering, typically by combining a strongly ferroelectric Pb(Zr,Ti)O_3_ (PZT) matrix with magnetically active perovskites such as Pb(Fe_0.5_Ta_0.5_)O_3_ (PFT), Pb(Fe_0.5_Nb_0.5_)O_3_ (PFN), or Pb(Fe_0.67_W_0.33_)O_3_ (PFW). This compositional tuning enables the deliberate coexistence of ferroelectric polarization, magnetic ordering, and magnetoelectric coupling at ambient temperature, making Pb‐based systems highly relevant for practical multiferroic applications [83, 84, 85].

One of the earliest and most thoroughly characterized systems is the solid solution (PbZr_0.53_Ti_0.47_O_3_)_(1‐x)_(PbFe_0.5_Ta_0.5_O_3_)_x_ (PZT‐PFT). For compositions in the range 0.2 ≤ x ≤ 0.4, room‐temperature multiferroicity has been demonstrated, where (PbZr_0.53_Ti_0.47_O_3_)_0.7_(PbFe_0.5_Ta_0.5_O_3_)_0.3_ exhibits well‐defined ferroelectric hysteresis loops at 300 K, with the remanent polarization of 16 µC cm^−2^ [83, 84]. At the same temperature, a magnetization value of 0.2 emu g^−1^ is obtained, arising from Fe‐related magnetic ordering within the perovskite lattice [83]. Linear magnetoelectric measurements reveal room‐temperature ME coefficients of 1.3 × 10e^−11^ s m^−1^, confirming intrinsic magnetoelectric coupling in this single‐phase material [83, 85]. Furthermore, the room‐temperature ME coupling was improved to 10^−7^ s m^−1^ in the same composition, attributed to the strain‐mediated coupling [86].

Another important Pb‐based family is Pb(Fe,M)_x_(Zr,Ti)_1‐x_O_3_ (PZT‐PFN), where M = Ta, Nb. For compositions with x = 0.2–0.35, the remanent polarization values typically lie in the range of 20–35 µC cm^−2^ at 300 K [85]. Magnetic measurements indicate cluster‐glass‐like behavior persisting at room temperature, with magnetization of 0.2 emu g^−1^, depending on Fe content and chemical disorder [85]. The corresponding room‐temperature linear magnetoelectric coefficients are typically on the order of 12 mV cm^−1^ Oe^−1^, as shown in Figure 6b [87]. A further notable series is (PbZr_0.52_Ti_0.48_O_3_)_1‐x_(PbFe_2/3_W_1/3_O_3_)_x_ (PZT‐PFW). For 0.2 < x < 0.4, PZT‐PFW exhibit stable room‐temperature remanent polarization up to 45 µC cm^−2^ and magnetization of 1.2 emu g^−1^ at 300 K [88]. The ferromagnetic response of PZT‐PFW can be significantly enhanced in their thin‐film form, reaching values on the order of 20 emu cm^−3^ [89]. Significantly, the linear ME coefficients of PZT‐PFW measured at room temperature can reach 20 mV cm^−1^ Oe^−1^ [88].

Aurivillius‐phase layered bismuth oxides constitute another important class of Type‐I multiferroic materials capable of exhibiting multiferroic functionality at room temperature. These compounds adopt a naturally layered perovskite structure consisting of perovskite‐like slabs separated by (Bi_2_O_2_)^2+^ layers, which inherently favors robust ferroelectricity. Magnetic ordering is introduced through partial substitution of transition‐metal ions (e.g., Fe, Co, Ni) into the perovskite blocks. Compared with BFO and Pb‐based multiferroics, Aurivillius phases offer advantages in terms of high thermal stability, low leakage currents, and strong ferroelectric polarization at ambient temperature.

Co/Fe co‐substituted bulk Aurivillius ceramics represent one of the earliest families of room‐temperature multiferroics. For example, Bi_3.25_Sm_0.75_Ti_3‐x_(Fe,Co)_x_O_12_ preserve a pronounced ferroelectric response, with a remanent polarization of 2 µC cm^−2^ at 300 K [90]. The magnetic behaviors are strongly dependent on dopant concentration, with magnetization values typically ranging from 0.2 to 0.6 emu g^−1^ [90]. In Sm‐ and Ni‐substituted compositions Bi_4‐x_Sm_x_Ti_3‐x_Ni_x_O_12_ (x = 0.02–0.07), a significantly larger room‐temperature remanent polarization of 9 µC cm^−2^ is obtained [91]. However, these ceramics exhibit only weak magnetization (0.005 emu g^−1^ at 300 K), associated with Ni‐containing perovskite layers. The linear ME measurements yield room‐temperature ME coefficients of 0.6 mV cm^−1^ Oe^−1^ (Figure 6c) [91]. Another noteworthy composition is Bi_4_NdTi_3_Fe_0.7_Ni_0.3_O_15_ ceramics, where Nd substitution on the A site is combined with Fe/Ni co‐occupancy on the B site to enhance both magnetic ordering and magnetoelectric coupling. These ceramics retain a remanent polarization of 4.3 µC cm^−2^ at ambient temperature, together with a magnetization of 0.2 emu g^−1^, Quantitative magnetoelectric measurements further reveal measurable magnetoelectric coupling between the ferroic order parameters, indicating that Fe/Ni co‐occupancy in the Aurivillius perovskite blocks can introduce magnetic functionality without fully suppressing the parent ferroelectric response [92].

The same design principles have been successfully translated into thin‐film geometries. Bi_6_Ti_2.8_Fe_1.52_Mn_0.68_O_18_ thin films are among the most extensively characterized Aurivillius multiferroics. Their ferroelectric properties have been confirmed by PFM, and the films exhibit a room‐temperature magnetization of 5 emu g^−1^ [93]. Moreover, magnetic‐field‐induced ferroelectric domain switching has been demonstrated in Bi_6_Ti_2.8_Fe_1.52_Mn_0.68_O_18_ thin films at room temperature [93], providing compelling evidence that the magnetoelectric coupling is intrinsic to the single‐phase layered structure rather than arising from large macroscopic magnetization or extrinsic composite effects. Another representative Aurivillius thin film is the four‐layered Bi_4_YFeTi_3_O_15_. At 300 K, they show clear ferroelectric hysteresis with remanent polarization of 27 µC cm^−2^ and a magnetization of 0.5 emu cm^−3^ [94]. Likewise, Bi_4_LaFeTi_3_O_15_ thin films exhibit stable ferroelectric polarization of 17 µC cm^−2^ at 300 K, together with a magnetization of 1.4 emu g^−1^. A strong ME coupling was observed at room temperature, with a converse ME coefficient of 21% [95].

Beyond BFO‐based, Pb‐based, and Aurivillius‐phase systems, a variety of structurally and chemically distinct compounds have also been reported to exhibit single‐phase multiferroicity at or near room temperature. Although these materials are generally less mature and less extensively characterized, they substantially broaden the materials landscape for ambient‐temperature multiferroics and introduce new structure–property paradigms.

Hexagonal LuFeO_3_ (h‐LuFeO_3_) has emerged as a prototypical non‐perovskite oxide multiferroic operating at room temperature. In epitaxial h‐LuFeO_3_ thin films, the coexistence of ferroelectric and magnetic order has been experimentally demonstrated at 300 K [96]. First‐principles and experimental studies predict ferroelectric polarization on the order of 5 µC cm^−2^, accompanied by weak ferromagnetism that persists at room temperature [97, 98]. Structural investigations confirm a noncentrosymmetric polar phase is stabilized in a metastable hexagonal lattice [97], while neutron and resonant x‐ray scattering measurements reveal antiferromagnetic order with a Neel temperature near or above room temperature [98]. The symmetry of the magnetic structure in the ferroelectric phase suggests that h‐LuFeO_3_ is a strong candidate for linear magnetoelectric coupling and for direct electric‐field control of the net ferromagnetic moment.

Complex oxides with unconventional structural motifs also display room‐temperature multiferroic behavior. For instance, epitaxial Bi_3_Fe_2_Mn_2_O_10_ thin films exploit intergrowth between BiFeO_3_ and BiMnO_3_ to stabilize a metastable Bi‐based supercell structure. This phase combines room‐temperature remanent polarization with ferrimagnetism, achieving a polarization of 10 µC cm^−2^ range and magnetization of 100 emu cm^−3^ [99]. This result highlights the ability of epitaxial strain and phase‐intergrowth engineering to create layered multiferroic architectures not accessible in bulk equilibrium phases. A more symmetry‐directed route is found in tilt‐engineered *n* = 2 Ruddlesden–Popper oxides, where layered perovskite slabs are separated by rock‐salt layers. Such as the system (1−x)(Ca_0.6_Sr_0.4_)_1.15_Tb_1.85_Fe_2_O_7_ – xCa_3_Ti_2_O_7_ (0.1 < x < 0.2) [100], octahedral rotations and tilts drive a polar distortion while also enabling spin canting and weak ferromagnetism above 300 K. The observation of a spontaneous polarization of 5 µC cm^−2^, together with weak magnetization persisting at room temperature, provide evidence that symmetry‐engineered lattice distortions can provide a common structural origin for polar and magnetic order in bulk layered oxides.

Gallium ferrite Ga_x_Fe_2‐x_O_3_ (GFO) is another well‐established room‐temperature multiferroic. In epitaxial GFO thin films (0 ≤ x ≤ 1), the coexistence of ferroelectric polarization and ferrimagnetic order has been demonstrated at 300 K, with polarization of 10 µC cm^−2^ and a magnetization of about 0.6 μ_B_ per formula unit [101, 102]. By adjusting the Fe concentration, both electric and magnetic properties can be systematically tuned while maintaining room‐temperature operation. The magnetoelectric coupling coefficient in GFO has been estimated to be 1.01 × 10^−3^ pm V^−1^ Oe^−1^. Dilute magnetic oxides can also exhibit multiferroic‐like behavior under specific conditions. For example, Cr‐doped ZnO thin films show room‐temperature ferroelectric‐like hysteresis and weak ferromagnetism, with polarization of 2 µC cm^−2^ and magnetization of 2.5 emu cm^−3^ at ambient temperature [103].

Beyond experimentally realized compounds, several materials have been theoretically predicted to exhibit multiferroic properties. First‐principles calculations predict near‐room‐temperature ferromagnetism and electric polarization as large as 10 µC cm^−2^ in R_2_NiMnO_6_/La_2_NiMnO_6_ heterostructures, where R is a rare‐earth ion, which exhibit an electrical polarization and strong ferromagnetic order near room temperature [104]. Moreover, first‐principles studies predict that hexagonal wurtzite MnSe (w‐MnSe) exhibits robust room‐temperature multiferroicity, combining sizable polarization and magnetic ordering in an altermagnetic framework, as shown in Figure 7a–f [105]. Although experimental realization is still pending, the predicted performance places w‐MnSe among the most promising emergent room‐temperature multiferroics. In addition, van der Waals multiferroics of the form M_2_XR_2_ (M = 3d transition metal, X = C or N, R = F or O) (Figure 7g–k) have been proposed to allow electrical control of magnetism near or above room temperature [106].

**FIGURE 7:**
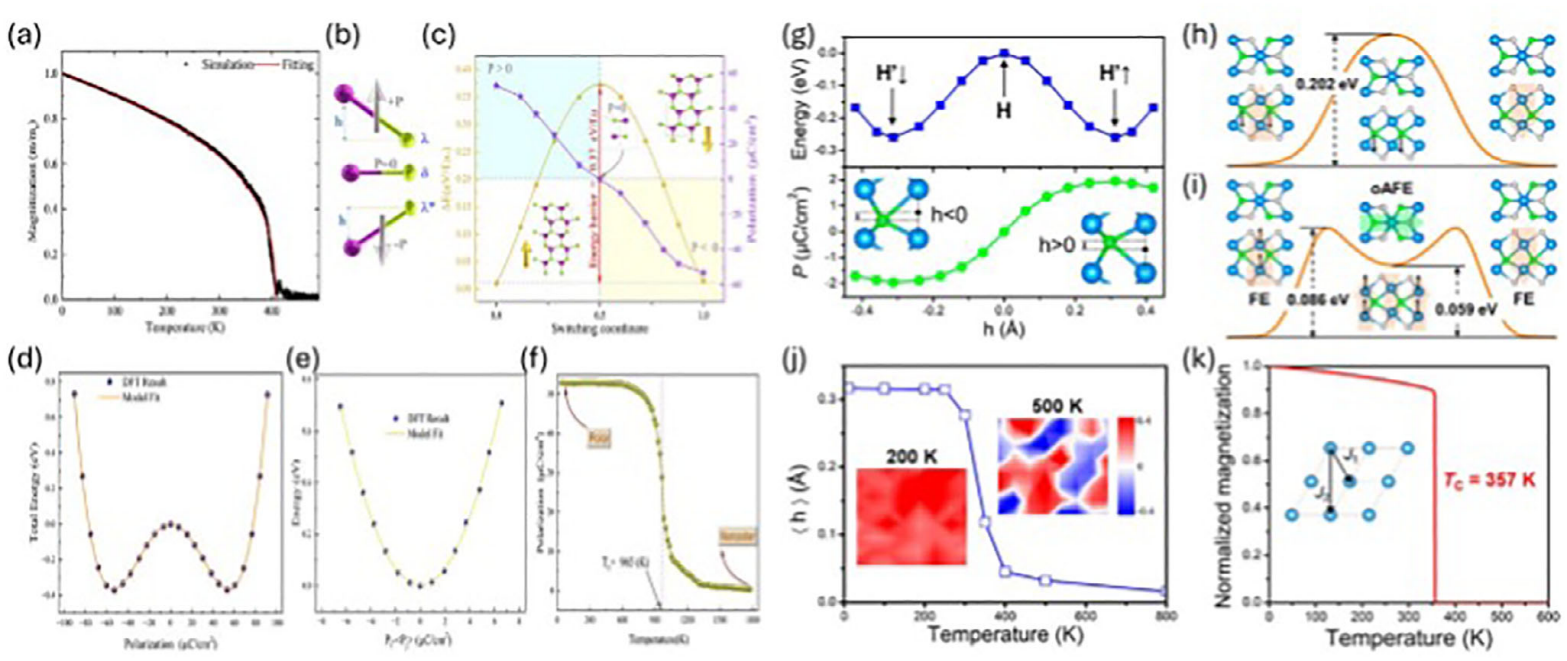
Polarization‐switching mechanisms and ferroelectric and magnetic properties of w‐MnSe and H′‐Co_2_CF_2_. (a) Temperature‐dependent sublattice magnetization of the w‐MnSe. (b) Schema of polarization switching mechanism: ferroelectric state (λ) and (λ*) with ± *P*_s_, paraelectric state with *P* = 0, where *δ* is the displacement of Mn ion relative to its position in the intermediate state. (c) The variation in spontaneous polarization along the z‐direction and the calculated energy barrier for ferroelectric Mn‐ion displacement in w‐MnSe. (d) Double‐well‐type variation of the energy for w‐MnSe as a function of polarization. (e) Dipole– dipole interaction calculated using mean‐field theory. (f) Monte Carlo simulation of temperature dependence of electric polarization [105]. (g) Total energy (upper panel) and electric polarization (lower panel) as functions of the displacement parameter h, which describes the geometric distortion of H′‐Co_2_CF_2_. Inset: the definition of h. (h, i) Two possible pathways for reversing the electric polarization. (j) Average value of h for an 8 × 8 × 1 supercell at different temperatures ranging from 10 to 800 K. Insets, snapshots of the distribution of h (ranging from −0.4 to 0.4 Å) for each Co–Co dimer in the 8 × 8 × 1 supercell at 200 and 500 K. (k) Calculated normalized magnetization as a function of temperature T [106].

In addition to inorganic systems, room‐temperature magnetoelectric effects have also been identified in molecular and hybrid multiferroics. A chiral lanthanide molecular ferroelectric based on a ytterbium(III) complex exhibits significant magnetoelectric coupling at room temperature, characterized by electrically switchable polarization and magnetic‐field‐dependent dielectric response [107]. Furthermore, first‐principles calculations combined with effective model Hamiltonians predict that two‐dimensional organic–inorganic systems such as Cr(h‐fpyz)_2_ (pyz = pyrazine) can display room‐temperature ferroelectricity coupled to magnetic order [108], demonstrating that multiferroicity is not limited to oxide‐based frameworks but can extend to a broader class of low‐dimensional and molecular materials.

Type‐I multiferroics offer a versatile materials platform for magnetoelectric technologies by combining ferroelectricity with magnetic order and allowing their coupled functionalities to be tailored through compositional, structural, and epitaxial engineering. In BFO, the interplay among ferroelectric polarization, oxygen‐octahedral rotations, and the Dzyaloshinskii–Moriya interaction provides an intrinsic route to electric‐field control of magnetic order at room temperature [5, 78, 79, 80, 81, 82]. This combination is particularly relevant to the voltage‐driven switching elements of MeRAM and MESO architectures, while the high ordering temperatures and thin‐film compatibility of BFO are favorable for thermal stability and nanoscale integration. Pb‐based and Aurivillius‐phase multiferroics complement BFO by offering additional flexibility in tailoring polarization, magnetization, magnetic ordering, and magnetoelectric coupling near room temperature [83, 84, 85, 86, 87, 88, 89, 91, 93, 95]. These characteristics broaden the materials basis for non‐volatile memory, magnetoelectric logic, and future multifunctional spintronic devices.

### 4.2 Type‐II Multiferroics

To date, hexaferrite compounds represent the most successful class of Type‐II multiferroics exhibiting robust multiferroic functionality at and above ambient temperature, while a small number of low‐dimensional systems have been proposed theoretically.

Hexaferrites with complex magnetic structures, particularly Z‐type and Y‐type hexaferrites, constitute the most technologically relevant Type‐II multiferroic materials operating at room temperature. The Z‐type hexaferrite Sr_3_Co_2_Fe_24_O_41_ is a prototypical example of this class (Figure 8a–g) [109, 110]. In polycrystalline Sr_3_Co_2_Fe_24_O_41_ ceramics, a clear magnetically induced ferroelectric polarization has been observed at 300 K (Figure 8h–l), originating from a transverse conical spin structure. The induced polarization is up to 5 µC m^−2^, and room‐temperature coefficients as large as 3 × 10^−9^ s m^−1^ (Figure 8m) [110]. Similarly, large linear ME coefficients on the order of 10^2^ ps m^−1^ have also been obtained in Y‐type hexaferrites such as BaSrCo_2_Fe_11_AlO_22_ [111].

**FIGURE 8:**
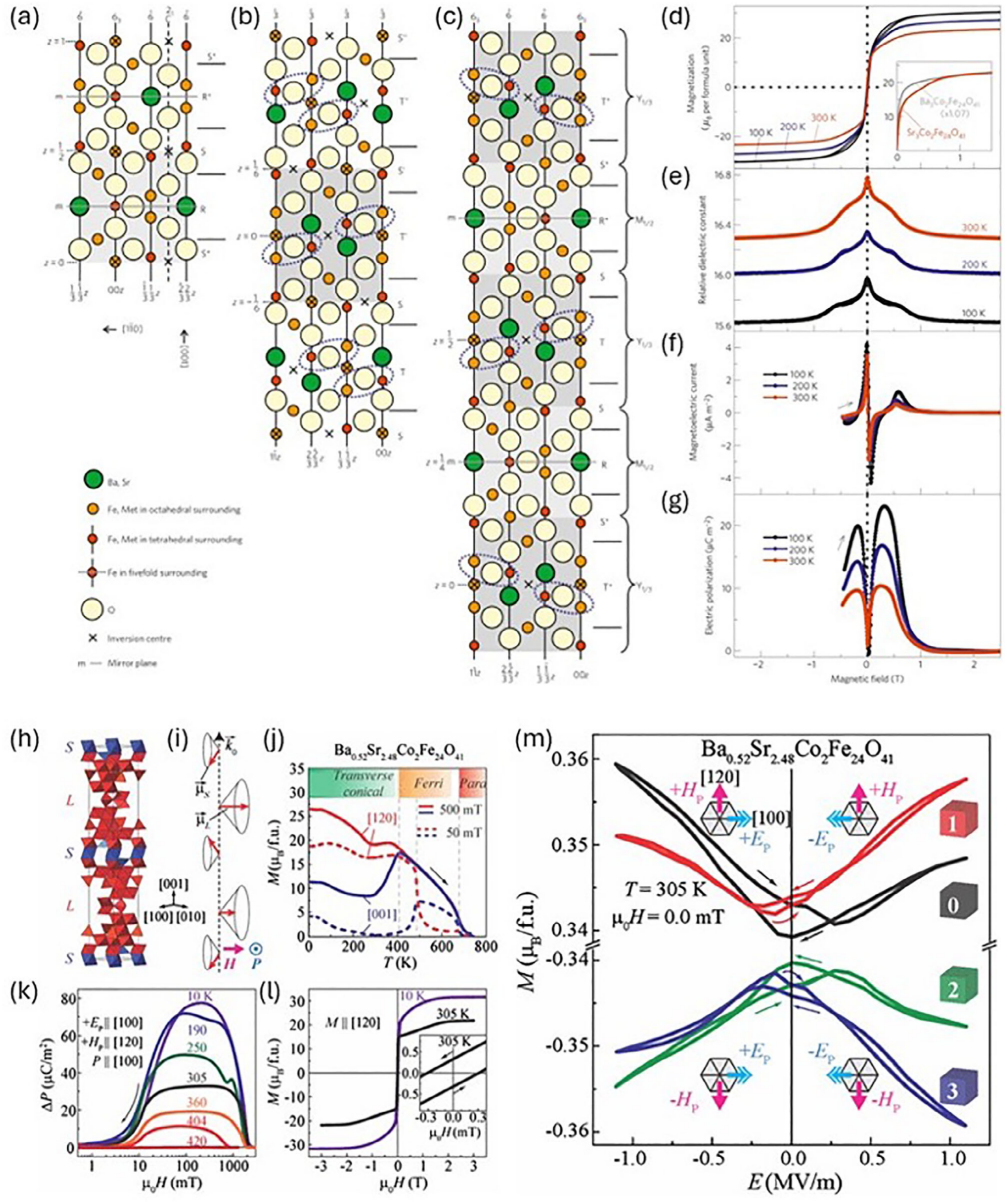
Structural and field‐dependent magnetoelectric properties of hexaferrites. The (110) cross‐section views of (a) M‐type [(Ba,Sr)Fe_12_O_19_], (b) Y‐type [(Ba,Sr)_2_Met_2_Fe_12_O_22_] and (c) Z‐type [(Ba,Sr)_3_Met_2_Fe_24_O_41_] structures with the hexagonal *c*‐axis vertical. Magnetic‐field B dependence of (d) magnetization, (e) relative dielectric constant, (f) magnetoelectric current and (g) electric polarization at 100, 200 and 300 K [109]. (h) The Z‐type hexaferrite structure in the hexagonal setting. (i) Schematic illustration of transverse conical spin states realized under a finite H. (j) Temperature dependence of M∥[120] and I∥[001]. (k) ΔP(*H*)( = P(*H*) − P(*H*_P_)) curves after ME poling with +μ_0_ *H*_P_ = 2 T (∥[120]) and +*E*_P_ = 230 kV/m (∥[100]). (l) M(*H*) curves at 10 and 305 K. The inset shows the magnified curve at 305 K. (m) The M(*E*) curves at zero *H* bias obtained after applying four different ME poling (states 0, 1, 2, and 3) as indicated in the inset [110].

Significantly, device‐level demonstrations have been realized in hexaferrites. Room‐temperature nonvolatile magnetoelectric memory elements have been fabricated using Sr_3_Co_2_Fe_24_O_41_ [26]. Operation at 300 K with stable retention and highly repeatable switching behavior confirms that hexaferrites are not merely laboratory curiosities, but genuinely viable Type‐II multiferroic materials for practical applications.

In addition to hexaferrites, low‐dimensional Type‐II multiferroics have attracted growing interest. First‐principles calculations predict that the Hf_2_VC_2_F_2_ MXene monolayer is a Type‐II multiferroic with a high magnetic transition temperature and magnetically driven ferroelectric polarization [112]. The predicted polarization is on the order of several µC cm^−2^, substantially larger than that of hexaferrites, with strong coupling between the magnetic and electric degrees of freedom. Such two‐dimensional systems delineate promising pathways for overcoming the polarization limitations of conventional Type‐II multiferroics while retaining high operating temperatures.

Type‐II multiferroics are particularly attractive for strongly coupled magnetoelectric memory and magnetic‐state control because their electric polarization is directly linked to the underlying magnetic order. This intimate coupling enables magnetic configurations to be transduced into electrically addressable states, providing a distinctive materials basis for non‐volatile magnetoelectronics. Room‐temperature hexaferrites can combine robust magnetic order, magnetically induced polarization, appreciable magnetoelectric coupling, and multistate magnetic functionality, making them especially promising for magnetoelectric memory and magnetic‐state storage [26, 109, 110, 111]. Their complex spin structures may also support multifunctional or multilevel operation, while low‐dimensional Type‐II materials offer an emerging pathway toward further miniaturization, enhanced polarization, and stronger coupling between electric and magnetic degrees of freedom [112]. These attributes could be valuable for future MeRAM and related magnetoelectric memory architectures.

### 4.3 Other Single‐Phase Multiferroics

In addition to canonical type‐I and type‐II multiferroics, a variety of other single‐phase materials have been shown to exhibit robust multiferroic behavior at or above room temperature. One representative example is the orthorhombic perovskite oxide LuFeO_3_, where the interplay of lattice distortions and canted antiferromagnetism yields a significant remanent polarization value of approximately 6 µC cm^−2^, together with a magnetization of 0.25 emu g^−1^ (Figure 9a,b) [113]. Also, double‐perovskite oxides such as Y_2_FeAlO_6_ (YFAO), which displays a remanent polarization of 0.04 µC cm^−2^ and a magnetization of 0.4 emu g^−1^ at room temperature, attributed to cation ordering and magnetic exchange interactions within the Fe─O network [114].

**FIGURE 9:**
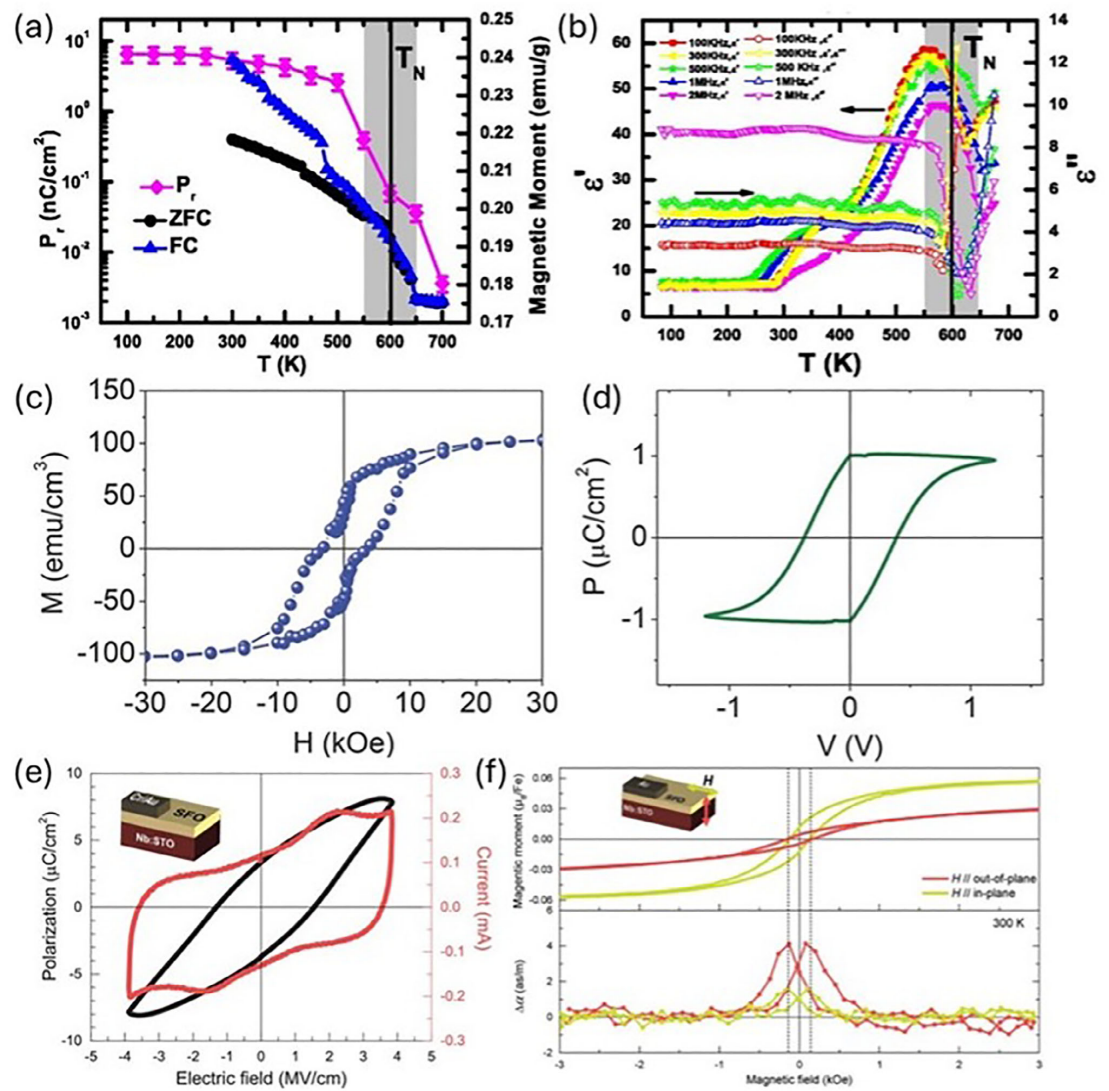
Ferroic and dielectric properties of LuFeO_3_, ε‐Fe_2_O_3_/AlFeO_3_ heterostructures, and SrFeO_2.5_ thin films. (a) The magnetization of LuFeO_3_ vs. temperature plots across 300–700 K showing the magnetic transition around ∼600 K (right *y*‐axis); measurements were carried out under ∼100 Oe field; also shown is the remanent polarization vs. temperature (left *y*‐axis); (b) real and imaginary dielectric permittivity of LuFeO_3_ vs. temperature patterns; an anomaly around ∼600 K is clearly visible in both the real and imaginary parts [113]. (c) Magnetization hysteresis loop at 300 K of ε‐Fe_2_O_3_/AlFeO_3_//Nb:STO(111) measured with the magnetic field applied in‐plane. (d) Ferroelectric hysteresis loop measured at 300 K and 10 Hz with 100 ms of delay time [115]. (e, f) Ferromagnetism of epitaxial SrFeO_2.5_ thin film magnetoelectrically coupled with electric polarization at room temperature [118].

Iron‐oxide‐based multiferroics constitute another important subclass. The metastable polymorph ε‐Fe_2_O_3_ has been shown to exhibit room‐temperature multiferroicity in thin‐film form (Figure 9c,d) [115]. At ambient conditions, ε‐Fe_2_O_3_ thin films display a remanent polarization of about 1 µC cm^−2^ and a magnetization of 100 emu g^−1^ [115, 116]. The simultaneous presence of ferroelectricity and strong magnetic anisotropy renders ε‐Fe_2_O_3_ particularly promising for high‐temperature and high‐frequency device applications. Related iron‐oxide and iron‐oxide‐derived compounds have likewise been investigated. For instance, β‐NaFeO_2_ has been identified as a room‐temperature multiferroic, exhibiting a polarization of ∼30 µC cm^−2^ along with weak ferromagnetism persisting up to 300 K [117]. Similarly, SrFeO_2.5_, which contains a one‐dimensional network of FeO_4_ tetrahedral chains, has been reported to host coexisting ferroelectric and ferromagnetic orders at room temperature, as shown in Figure 9e,f [118].

Furthermore, (Fe_x_,Sr_1‐x_)TiO_3_ thin films have also been demonstrated to exhibit room‐temperature multiferroicity for suitable Fe concentrations [119]. At 300 K, these films show remanent polarization values on the order of 0.5 µC cm^−2^, while magnetic measurements reveal clear ferromagnetic behavior with magnetization values approaching 60 emu cm^−3^, depending on the Fe content.

Emerging non‐canonical single‐phase multiferroics extend the design space through cation ordering, non‐centrosymmetric structural distortions, epitaxial stabilization, metastable phase formation, layered structural motifs, chemical substitution, and dimensional reduction. These strategies enable access to combinations of room‐temperature polarization and magnetic order with enhanced magnetic anisotropy, high transition temperatures, relatively large magnetization, and unconventional structural functionality in selected systems [96, 98, 100, 102, 105, 108, 116, 118]. Such characteristics are attractive for thermally robust magnetoelectric memory, voltage‐controlled magnetic switching, high‐frequency magnetoelectric devices, and future magnon‐based architectures. In particular, antiferromagnetic materials provide a promising basis for magnonic functionality through their rapid spin dynamics, low stray fields, and favorable scaling characteristics when incorporated into appropriately engineered device structures, providing a potential materials basis for expanding the functional range of MeRAM, MESO, and magnon‐based magnetoelectronics.

A comparison of these material classes reveals a fundamental balance between ferroic robustness and magnetoelectric responsiveness. Materials with strong polar distortions generally offer larger and more stable ferroelectric polarization, whereas strong magnetoelectric coupling is often associated with specific magnetic configurations, structural distortions, and low‐symmetry lattice states. Chemical substitution, cation ordering, epitaxial strain, layered architectures, and dimensional reduction provide routes to modify this balance by regulating polarization, magnetic order, magnetic anisotropy, and the coupling between electric and magnetic degrees of freedom. The resulting materials landscape therefore spans robust room‐temperature ferroic behavior, strong magnetic–electric coupling, electrically controllable magnetic states, and enhanced magnetic or thermal stability.

This balance also determines the potential function of a material within a magnetoelectronic device. Materials that combine stable polarization with efficient electric‐field control of magnetic order are well suited to the switching element of MeRAM and MESO, whereas materials possessing complex antiferromagnetic order and favorable spin dynamics are more relevant to magnon‐mediated information transport. Device‐relevant materials will therefore require the coordinated optimization of the microscopic coupling mechanism, magnetic configuration, switching pathway, and thin‐film properties. At the device level, translating these material characteristics into reliable functionality further requires control over interfaces, readout processes, signal transduction, and integration with scalable device architectures.

The materials‐to‐device relationship provides the basis for assessing the technological relevance of different multiferroic systems beyond their individual polarization, magnetization, or magnetoelectric coefficients. The transition from materials‐level functionality to practical magnetoelectronic technologies ultimately depends on preserving the coupled ferroic response during thin‐film growth, device fabrication, and integration. These considerations motivate the discussion of manufacturing, interfacial, and architectural challenges in the following section.

## 5 Manufacturing, Integration and Architectural Challenges

In modern integrated circuits, reliability, reproducibility, and compatibility with large‐scale, high‐throughput fabrication remain enduring and unavoidable concerns in practical manufacturing, alongside device performance itself. These requirements distinguish practical manufacturing sharply from laboratory‐scale demonstrations, where results are often established on a limited number of optimized devices.

In general, the pathway toward manufacturability may be evaluated at three levels, as illustrated in Figure 10a. First, beyond demonstrating competitive performance, the reliability of individual devices must be established through application‐relevant metrics, such as threshold‐voltage stability, leakage stability, retention, endurance, or cycle‐to‐cycle reproducibility, depending on the target device function. Second, these same metrics must be assessed statistically across devices fabricated on the same wafer to determine device‐to‐device variation, uniformity, and yield. Third, the evaluation must be extended across multiple wafers to examine wafer‐to‐wafer reproducibility, while broader manufacturing considerations, including throughput and cost, also become critical. Across these three levels, the key challenges governing the manufacturability of emerging logic and memory devices can generally be classified into three broad categories: thin‐film growth, device fabrication, and integration with CMOS or established device stacks.

**FIGURE 10:**
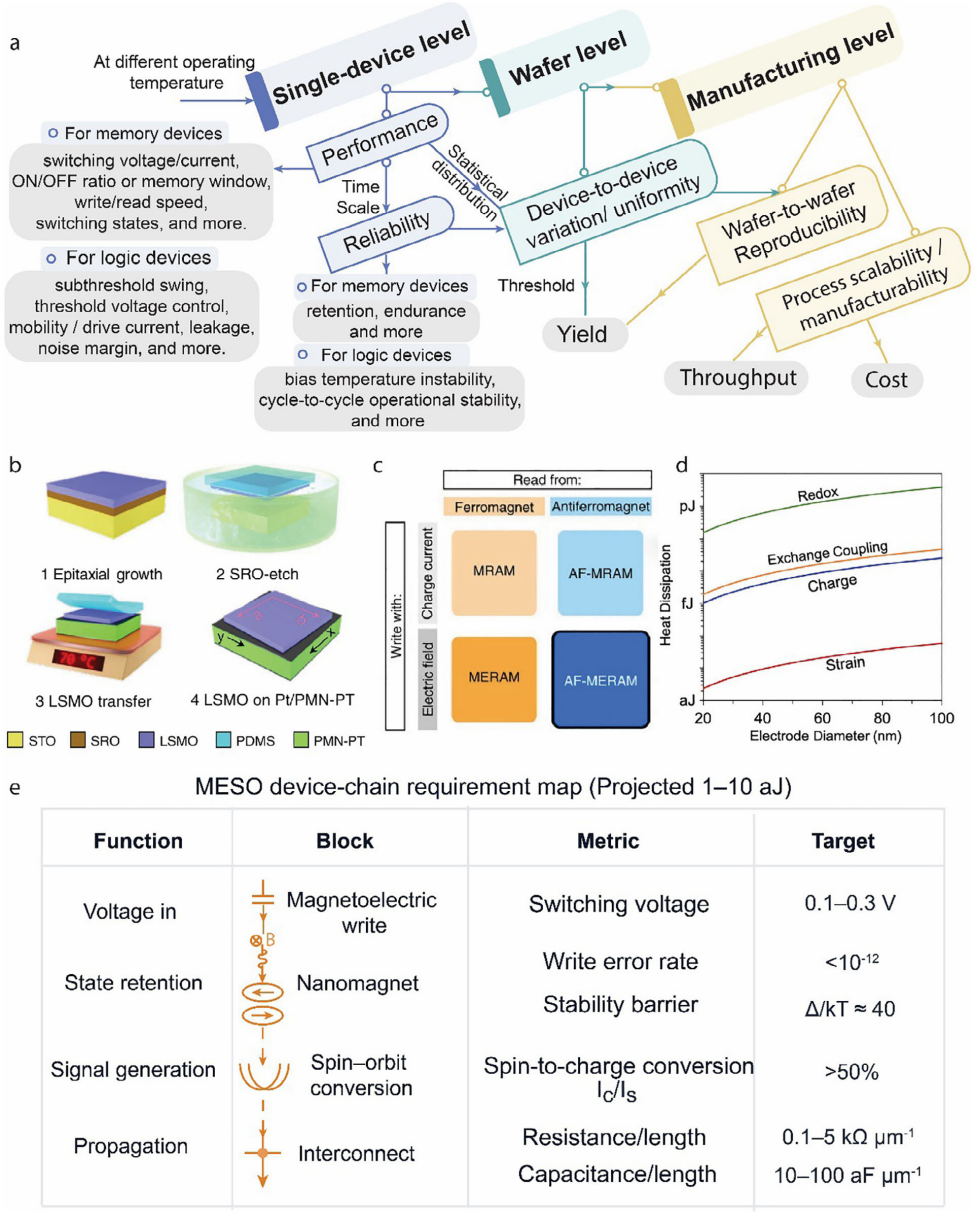
Manufacturing, integration and architectural challenges. (a) Schematic framework illustrating the pathway from proof‐of‐concept devices to practically deployable technologies, and progression from single‐device demonstration to manufacturing readiness for emerging logic and memory devices. At the single‐device level, key considerations are performance and reliability. At the wafer level, the focus shifts to device‐to‐device variation, statistical validation, and yield. At the manufacturing level, the major challenges become wafer‐to‐wafer reproducibility, integration compatibility, scalability/manufacturability, throughput, and cost. (b) Four‐step release‐transfer process for integrating artificial multiferroic heterostructures [120]. (c) Classification of non‐volatile magnetic random‐access memory elements by write and read scheme, illustrating the multiple stack combinations possible in mERAM and the resulting need for write–read co‐design [27]. (d) Calculated total heat dissipation per switching event for representative ME switching mechanisms as a function of electrode diameter, highlighting the projected reduction in switching dissipation with lateral scaling and the potential for ultralow‐energy operation in selected ME device concepts [121]. (e) Key device and material requirements for enabling 1–10 aJ‐class MESO logic [8].

Thin‐film growth is particularly constrained by both fundamental and engineering limitations. A persistent tension exists between the need for precise control of film quality and the demands of scalable manufacturing, especially as films are pushed toward smaller thicknesses while fabrication is extended over larger areas and higher throughput. At the fundamental level, this challenge is reflected in the trade‐off between slow, highly controlled growth and the achievement of high‐quality thin films. At the engineering level, further difficulties arise in maintaining uniform growth conditions across large areas, including temperature uniformity, environmental stability, and homogeneous delivery of source materials.

When moving beyond film‐level demonstrations to practical device fabrication, further challenges emerge from processing steps that can perturb thin‐film properties and become increasingly difficult to control at aggressively scaled dimensions. Such steps include lithography, etching, metallization, annealing, and encapsulation. For instance, lithographic proximity effects, etch‐induced sidewall roughness, debris redeposition, and non‐conformal metal coating may be tolerable in larger‐scale devices, yet in ultrascaled nanometre structures even atomic‐scale variations can produce significant device‐to‐device variability. Consequently, the challenge is no longer limited to obtaining high‐quality films, but extends to preserving functional uniformity across both device arrays and entire wafers.

Beyond fabrication itself, successful integration with CMOS or existing device stacks places additional demands on process compatibility. Thermal budget constraints imposed by surrounding materials and components may limit the temperatures available for film growth or post‐deposition annealing, even though such conditions are often required to achieve high‐quality films. At the same time, device functionality and reliability must remain stable through back‐end‐of‐line integration steps, including wafer bonding and related processing sequences. Together, these constraints further complicate the practical deployment of advanced thin‐film devices in integrated electronic systems.

Although these constraints already complicate the practical deployment of advanced electronics in integrated systems more broadly, multiferroic devices face additional and more distinctive challenges. In the following sections, we therefore examine the specific manufacturing, integration, and architectural barriers that must be overcome for multiferroic devices to achieve practical technological relevance.

### 5.1 Multiferroic Engineering: Theory vs. Production Reality

Single‐phase multiferroics are attractive because they integrate coupled ferroic functionalities within a single material phase, offering a conceptually compact route toward non‐volatile and potentially low‐power devices. However, this intrinsic integration also makes them highly manufacturing‐sensitive: multiple coupled order parameters must be stabilized simultaneously under tightly controlled compositional, structural, and defect conditions [122]. In essence, the closer magnetoelectric coupling lies to intrinsic materials physics, the harder it becomes to preserve through practical manufacturing [123].

This creates unusually stringent requirements on phase purity, stoichiometric control, defect suppression, oxygen‐vacancy concentration, and structural uniformity, all of which strongly influence ferroic ordering, leakage behavior, polarization switching, and magnetic response [124, 125]. Functional performance is further tied to domain configuration and strain‐mediated distortions that are highly sensitive to processing history [10, 126, 127]. As a result, reproducible manufacturing remains challenging because the desired electric and magnetic responses must be maintained simultaneously within a narrow process window. In practice, even seemingly minor process variations that might be tolerable in conventional fabrication can be difficult to control consistently across batches, laboratories, and manufacturing environments at sustainable cost.

Encouragingly, recent progress suggests that manufacturability and functionality need not be mutually exclusive. For example, low‐temperature La‐substituted BiFeO_3_ heterostructures have shown that oriented multiferroic oxide films can be grown at substantially reduced thermal budgets while retaining switchable ferroelectric behavior, long retention and fatigue endurance [128]. This is significant because conventional high‐quality BiFeO_3_‐based films typically require high‐temperature epitaxial growth, which limits compatibility with back‐end or heterogeneous integration. In parallel, La substitution has been shown to improve polarization switching and fatigue behavior in BiFeO_3_ thin films [129], while atomic‐layer‐controlled deposition routes provide a route to improved thickness, stoichiometry and interface control at lower growth temperatures [130, 131]. These developments suggest that the key challenge is shifting from simply discovering new multiferroic compounds toward engineering process‐tolerant oxide platforms that preserve sufficient ferroic functionality under realistic deposition, integration, and manufacturing constraints.

### 5.2 Interfacial Hurdles in Magnetoelectric Composites

Compared with single‐phase multiferroics, engineered magnetoelectric platforms provide versatile options for realizing highly functional devices. However, manufacturing‐related challenges have shifted the focus toward interface engineering and heterostructure compatibility [3]. The central difficulty lies in reproducibly integrating multiple functional materials into a low‐defect heterostructure that remains chemically stable, structurally coherent, and efficiently coupled across interfaces [8].

The performance of multilayer structures is ultimately governed by their interfaces, which mediate coupling between the different functional layers [132, 133]. Interfacial roughness, interdiffusion, charge trapping, and inefficient strain transfer can therefore severely compromise the intended magnetoelectric response, even when the individual layers exhibit strong standalone performance [134, 135]. These issues may be further aggravated by device fabrication steps such as etching, metallization, and annealing, which can disturb delicate multilayer interfaces. In practical devices, additional interfaces beyond the main coupling layers, including ferroelectric/electrode, ferromagnet/dielectric, oxide/semiconductor, and multiferroic/heavy‐metal or tunnel‐barrier interfaces, introduce further non‐idealities such as dead layers, current leakage, fatigue, depolarisation, and spin scattering.

These interface effects become particularly important in magnetoelectric logic architectures that rely on spin‐to‐charge conversion for readout and signal generation [8]. In representative CoFe/Pt readout devices, a spin‐polarized current injected from CoFe into Pt is converted through the inverse spin Hall effect and collected as a transverse spin–orbit voltage [9, 10]. However, the efficiency of this transduction is strongly limited by the ferromagnet/spin–orbit‐material interface. During spin injection and conversion, a substantial fraction of spin angular momentum may be reflected, scattered, or depolarized owing to finite spin‐mixing conductance, spin‐memory loss, conductance mismatch, interfacial spin–orbit scattering, chemical intermixing, and structural disorder [136]. Consequently, the effective spin transparency is reduced, so only part of the generated spin signal contributes to the measurable output voltage.

CoFe and CoFeB interfaces with heavy metals such as Pt, Ta or W, as well as ferromagnet/topological‐insulator interfaces, have therefore become model systems for identifying interfacial intermixing, proximity‐induced magnetism and degraded interfacial electronic structure as key sources of signal loss [137, 138]. Improving spin transparency through atomic‐scale interface control, insertion or spacer layers, suppressed intermixing, structural matching and optimized interfacial electronic alignment therefore remains a critical requirement for efficient spin–orbit transduction and practical magnetoelectric logic operation [138, 139].

Moreover, interfaces should not be viewed solely as a source of loss. Increasingly, they are being exploited as a means of enhancing device functionality. Advances in epitaxial growth, insertion‐layer engineering, interfacial oxidation control, and topological‐material‐based spin–orbit transducers have demonstrated substantial improvements in spin transmission, spin–charge conversion efficiency, and interfacial coupling strength. These developments highlight the growing role of interface design as an active tool for performance optimization rather than merely a fabrication constraint.

A further bottleneck arises from the incompatibility of growth and processing windows across the heterostructure. For example, a ferroelectric layer may require oxidation‐rich or high‐temperature processing, whereas a neighboring magnetic layer may be vulnerable to oxidation, intermixing, or thermal degradation. Thus, device integration often becomes a compromise between preserving individual material quality and achieving stack‐level process compatibility [140]. Novel integration strategies, such as epitaxial lift‐off and transfer Figure 10b, have enabled the assembly of high‐quality artificial multiferroic heterostructures with large magnetoelectric coupling [120]. However, their translation toward practical device implementation remains at an early stage.

Taken together, these considerations illustrate a broader shift in the field. The central challenge is no longer the demonstration of magnetoelectric coupling itself, but the ability to preserve, transmit and exploit that coupling across increasingly complex device architectures. As a result, advances in interface engineering are becoming as important as advances in the underlying multiferroic materials, providing one of the clearest pathways toward practical magnetoelectronic memory and logic technologies.

More broadly, the practical challenge in multiferroic devices lies not simply in establishing magnetoelectric coupling, but in integrating switching and readout or output generation into a reproducible device platform capable of supporting useful memory or logic functionality [27]. In MERAM, for instance, multiple combinations of write and read schemes have been proposed, as summarised in Figure 10c. This diversity makes co‐design of the write and read stacks an important practical consideration, while introducing further constraints associated with interface quality, write–read decoupling, sensing contrast, and read disturbance. Similarly, in MESO, low‐voltage and reproducible magnetoelectric switching must be coupled to efficient spin–orbit conversion of the switched magnetic state into a usable electrical output, making conversion efficiency, parasitic losses, and interface quality key operational bottlenecks [10]. ME devices exhibit strong promise for ultralow‐energy operation, with switching energies projected to decrease into the attojoule regime as the lateral dimensions are reduced, as shown in Figure 10d [121]. However, achieving these projected benefits, particularly in terms of energy efficiency, requires the simultaneous optimization of all device components, rather than the performance of any single core element in isolation, as illustrated in Figure 10e using MESO as an example [8].

### 5.3 Architectural Viability and Benchmarking Beyond CMOS

At the architectural level, a low‐energy switching event at the device level does not necessarily imply a low‐energy chip‐level implementation. The key question is no longer whether coupled ferroic behavior can be demonstrated, but whether it can enable a computing paradigm that offers measurable advantages over established CMOS‐based systems. This distinction is critical because attractive device‐level figures of merit, such as low switching energy, projected write voltage, or non‐volatility, do not automatically translate into architectural benefit. For any logic family (like CMOS or MESO) to be useful, the output signal of one gate must be strong enough to trigger the input of the next gate (or multiple gates) [6]. This is known as Fan‐out. Currently, the signal generated by the “reading” process (Spin‐to‐Charge conversion) is often too weak [43]. If the output voltage is lower than the required switching voltage of the next device, the signal dies, and the circuit fails. A recent MESO readout study states this problem very directly: the output voltage from the spin‐to‐charge conversion readout is still **far below the minimum magnetoelectric writing voltage** needed for the next stage. This study notes that while magnetoelectric writing has been pushed to about **150 mV**, with a pathway toward **100 mV**, the experimentally obtained readout voltage is often only **less than 20 microvolts,** leaving an enormous gap between what the device can read out and what the next device needs to switch [56]. Similarly, interconnect overheads, sensing circuitry and peripheral electronics can contribute significantly to the overall energy budget, potentially eroding the advantages suggested by isolated device metrics. Meaningful benchmarking therefore requires evaluation at the circuit level, where device performance must be considered alongside signal propagation, latency, area efficiency and interconnect costs.

Architectural considerations further amplify the importance of reliability and variability. Material‐ and device‐level non‐idealities that appear minor in isolated demonstrations can propagate across arrays or cascaded logic networks in the form of write failure, read ambiguity, threshold broadening, drift, retention spread, or stochastic switching [13]. Their consequences are strongly application‐dependent. Memory arrays are particularly sensitive to retention, endurance, and disturb effects, whereas logic circuits require deterministic operation, fan‐out, and robust cascading [141]. Neuromorphic systems may tolerate a degree of stochasticity, but remain vulnerable to drift, nonlinearity, asymmetry, and device variability [142]. Thus, the architectural tolerance to device imperfections is not universal, but depends strongly on the intended computational role of the device [143].

Device scaling introduces additional complexity. Although reducing device dimensions can improve integration density and, in principle, lower switching energies toward the attojoule regime, the accompanying reduction in functional volume may compromise state stability, sharpen the trade‐off between switchability and thermal stability, and increase the probability of write errors [121]. At the same time, thinner functional layers become increasingly susceptible to leakage, interfacial non‐idealities, and statistical variability [144]. Moreover, higher array density can intensify practical dissipation and thermal‐management constraints. As a result, cell‐to‐cell variation, switching distributions, sensing margin, and compatibility with access or selector circuitry become critical determinants of array‐level feasibility [145].

A deeper architectural issue lies in the gap between novel devices and unconventional computing paradigms. The challenge is not simply how to interconnect emerging devices, but how to exploit their unique state variables and operating principles within a meaningful computational framework [1, 146]. Rather than serving as direct transistor replacements, many multiferroic and magnetoelectric device concepts are more naturally aligned with stateful logic, in‐memory computing, majority logic, or spin‐based signal representation. Their practical adoption may therefore require rethinking logic style, signal encoding, write/read protocols, circuit design methodology, and electronic design automation support [145, 147]. Viewed from this perspective, the architectural challenge is also the greatest opportunity: exploiting the unique properties of magnetoelectric devices may require rethinking not only the device, but the organization of computation itself.

## 6 Research Priorities for Magnetoelectronic Memory and Logic

The challenges outlined in the preceding sections suggest that the future of magnetoelectronic memory and logic will depend less on discovering entirely new physical phenomena than on translating existing magnetoelectric effects into reliable, manufacturable, and architecturally relevant technologies. Across materials, devices and computing systems, several priorities emerge that are likely to determine whether magnetoelectric concepts can progress from laboratory demonstrations to practical computing platforms.

These priorities span multiple levels of the technology stack, ranging from materials and interfaces to devices, circuits and computing architectures. Progress at any individual level is unlikely to be sufficient in isolation; rather, advances must occur simultaneously across the hierarchy illustrated in Figure 11.

**FIGURE 11:**
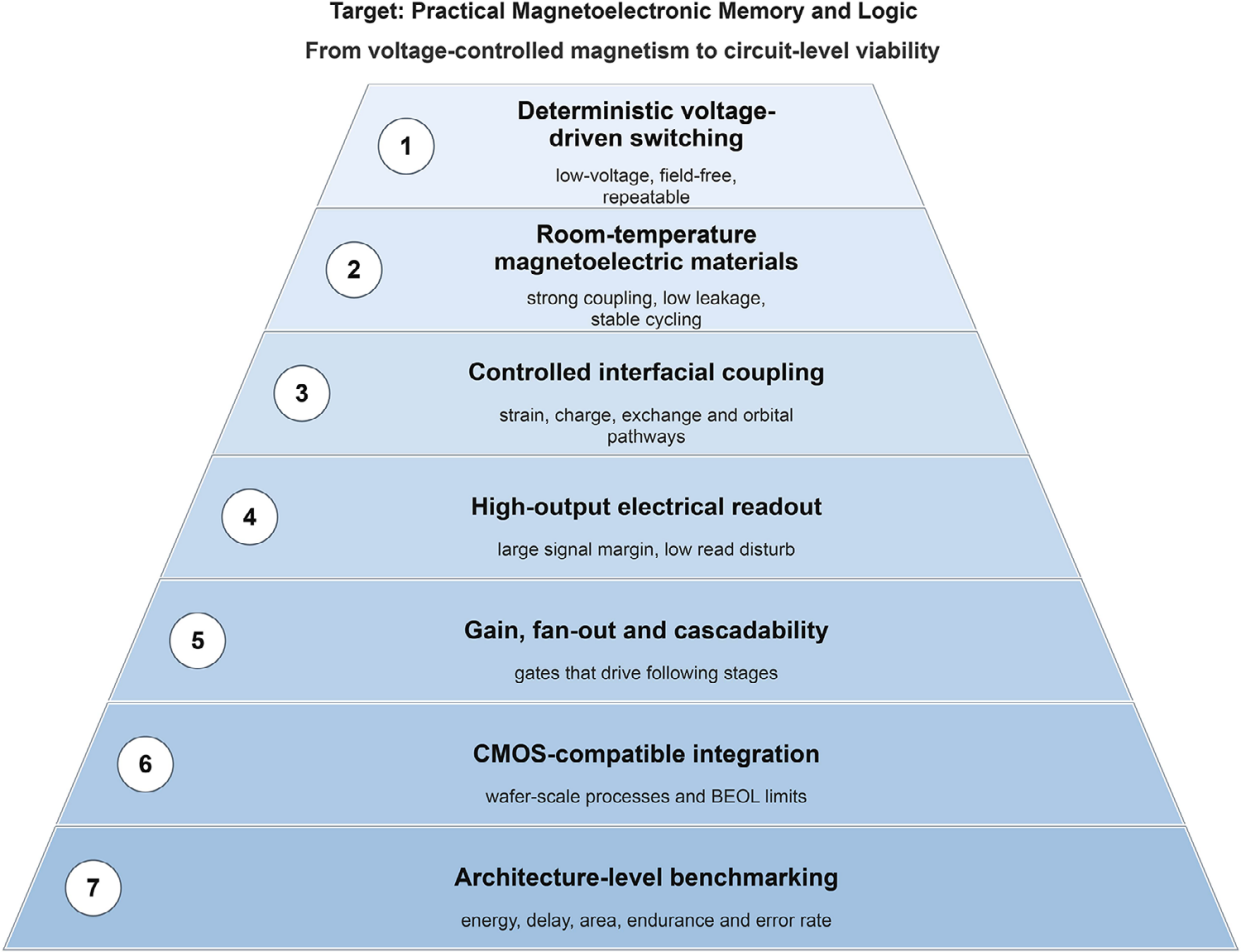
Research priorities for Magnetoelectronic Memory and Logic.

At the materials level, the challenge is to simultaneously achieve room‐temperature operation, strong magnetoelectric coupling, low leakage, high endurance, and compatibility with thin‐film processing. Although Type‐I and Type‐II multiferroics provide complementary advantages, no existing material system fully satisfies all device requirements. Consequently, future advances are likely to depend not only on discovering new multiferroics but also on engineering compositions, defects, and heterostructures that expand the accessible design space.

At the device level, deterministic voltage‐driven switching remains the most immediate requirement. Although electric‐field control of magnetism has now been demonstrated in numerous material systems, practical logic and memory applications require switching that is reproducible, field‐free, and compatible with operating voltages approaching the 100 mV regime. Encouragingly, recent progress in VCMA‐based devices, exchange‐coupled heterostructures and magnetoelectric switching has substantially reduced switching voltages. The remaining challenge is to achieve deterministic operation while maintaining thermal stability, endurance, and scalability.

Interfaces represent one of the most promising opportunities for performance improvement because many of the current limitations originate at the atomic scale. Charge‐mediated coupling, strain transfer, exchange bias, and spin–orbit conversion are all governed by interfacial phenomena. Consequently, advances in epitaxial growth, insertion‐layer engineering, oxidation control, and topological‐material‐based transducers have already demonstrated substantial improvements in coupling strength and spin‐transparency. Future work should increasingly treat interfaces as active functional components rather than passive boundaries, with the goal of engineering interfacial electronic structure to maximize both magnetoelectric coupling and signal generation.

Equally important is the development of efficient readout mechanisms. Recent demonstrations combining magnetoelectric writing with electrical spin–orbit readout represent important progress because they address a long‐standing gap between switching and signal generation. Nevertheless, output voltage, gain, and fan‐out remain limiting factors. Improving spin‐to‐charge conversion efficiency through optimized interfaces, heavy‐metal transducers and topological materials may provide a pathway toward practical cascaded logic operation.

Looking forward, the most likely path for magnetoelectronic technologies is not a direct replacement of CMOS transistors, but a staged progression from materials optimization to architectural innovation. In the near term, efforts should focus on deterministic switching, interface engineering, and low‐voltage operation. Over the next decade, priorities are expected to shift toward CMOS‐compatible integration, signal transduction and circuit‐level demonstrations. Ultimately, the greatest impact of multiferroics may emerge from computing paradigms that exploit their intrinsic non‐volatility and voltage‐controlled operation, including logic‐in‐memory, compute‐near‐memory and normally‐off computing. In this sense, the future of magnetoelectronics will be defined not only by advances in materials and devices, but also by the ability to co‐design architectures that leverage functionalities unavailable to conventional CMOS technology.

## Author Contributions

**Yang Liu**: data curation, writing – review and editing, writing – original draft, conceptualization, supervision. **Yunlong Sun**: writing – original draft, writing – review and editing, data curation. **Hao Luo**: writing – original draft, writing – review and editing, data curation. **Duoduo Zhao**: data curation. **Yuhan Zeng**: data curation. **Xuzihan Zhang**: data curation. **Zhewei Liu**: data curation. **Hao Peng**: data curation. **Zhijing Zhang**: data curation. **Jack Yang**: writing – review and editing. **Ji Zhang**: writing – review and editing. **Danyang Wang**: writing – review and editing. **Junjie Shi**: writing – review and editing, data curation. **Sean Li**: writing – review and editing.

## Conflicts of Interest

The authors declare no conflicts of interest.

## Data Availability

Data sharing is not applicable to this article as no datasets were generated or analyzed during the current study.
